# *Micromonas* , a small pigmented flagellate, predominates the nanoflagellate and photosynthetic picoeukaryote communities in the northern South China Sea

**DOI:** 10.1111/1758-2229.13244

**Published:** 2024-03-27

**Authors:** Xin Guo, Mengwen Pang, Xinyi Zheng, Lingfeng Huang

**Affiliations:** ^1^ Key Laboratory of the Ministry of Education for Coastal and Wetland Ecosystems, College of the Environment and Ecology Xiamen University Xiamen China; ^2^ Department of Ocean Science Hong Kong University of Science and Technology Hong Kong China

## Abstract

A small pigmented flagellate, *Micromonas*, is prevalently distributed in coastal and pelagic waters. However, there have been few studies conducted to quantify their abundance in the marginal seas of the Northwest Pacific Ocean. In this study, we used fluorescent in situ hybridization with tyramide signal amplification (TSA‐FISH) to reveal the spatial distribution of *Micromonas* in the northern South China Sea (SCS). On average, the abundance of *Micromonas* was 317 cells mL^−1^, with the average proportions in the nanoflagellates (NF) and photosynthetic picoeukaryotes (PPE) communities being 10.94% and 15.39%, respectively. This indicates a wide distribution and dominance of this genus in the studied area. The relationships between *Micromonas* abundance and various environmental factors suggested that biotic correlations play more important roles than physicochemical filtering on *Micromonas* assemblage. This may indicate a broad environmental adaptation spectrum of this genus through its flexibility in terms of resource acquisition strategies. In summary, this study provides insight into the spatial distribution pattern of *Micromonas* and highlights its crucial contribution to the composition of NFs and PPE communities, which rely on biological interaction to respond to the changing environmental conditions in the northern SCS.

## Graphical abstract

Employing fluorescent in situ hybridization with tyramide signal amplification (TSA‐FISH), we specifically detected and quantified the genus Micromonas. This genus exhibited a vertical distribution across both nearshore and offshore habitats in the northern South China Sea, forming a significant proportion of the microbial community. The prevalence and dominance of *Micromonas* may be attributed to its flexible mixotrophic strategy, where both inorganic nutrients and food sources (such as bacteria) play crucial roles in its assembly.

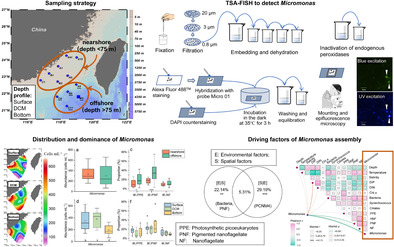

## INTRODUCTION

Nanoflagellates (NFs) are widespread unicellular protists with a cell size of 0.8–20 μm that move and prey using their flagella (Throndsen, 1997). Based on the presence of pigments, NFs can be divided into heterotrophic NFs (HNFs) and pigmented NFs (PNFs). The former are known to be the major consumer of bacteria, which not only regulate prey density but also their community composition (Pernthaler, 2005). PNFs on the other hand are significant contributors to marine primary production, as well as the phytoplankton biomass (Crespo et al., 2011). However, mixotrophy of PNF has also been described in the oligotrophic zone (Zubkov & Tarran, 2008) or in areas where nutrients are abundant (Figueiras et al., 2020), meeting their metabolic demands through a mixed trophic mode that combines both photosynthesis and prey ingestion, which are known as mixotrophic NFs (MNFs; Caron, 2016; Flynn et al., 2019). Studies have found that MNFs make up a large proportion of PNFs, for example, about 50% in the Sargasso Sea (Arenovski et al., 1995) and the NW Mediterranean (Unrein et al., 2014), 5%–10% in the Ross Sea (Moorthi et al., 2009), 2%–29% in a coastal upwelling area in Chile (Vargas et al., 2012) and 5%–36% in the *Tara* Oceans project (Li et al., 2022), significantly impact bacterioplankton dynamics via grazing (Stoecker et al., 2017). Mixotrophic strategies give competitive advantages to different mixotrophs relative to their strict auto‐ and hetero‐trophic competitors when a single nutritional mode does not fulfil cellular nutrient requirements (Leles et al., 2018; Mansour & Anestis, 2021). As an organism's ability to use alternative forms of nutrition, mixotrophy plays an important role in the energy budgets and biogeochemical cycling in planktonic ecosystems (Stoecker et al., 2017). On the one hand, autotrophic mixotrophs can involve phagotrophy to supplement a variety of cellular nutritional needs including the acquisition of C, N, P and other nutrients, thus contributing more to marine primary production (Wilken et al., 2014). On the other hand, phototrophic behaviour incorporated in consumers could enhance their gross growth efficiency to a certain extent, whose feeding and metabolism processes are often accompanied by the regeneration of nutrients, which can be reutilized by autotrophs for further primary production (Sherr & Sherr, 2009). Therefore, the overall effect of mixotrophy is to boost the trophic transfer efficiency from microbial loop to larger size classes of plankton in the marine ecosystem compared with the situation where only strict autotrophy and heterotrophy exist (Stoecker et al., 2017); and thereby improve the efficiency of the microbial C pump that leads to C storage in the deep ocean (Jiao et al., 2010; Selosse et al., 2017). Photosynthetic picoeukaryotes (PPE), with cell sizes less than 3 μm and slightly larger than cyanobacteria, have been observed to typically dominate 60%–80% algal biomass and primary production globally (Massana, 2011), appearing as the important contributors to picoplankton community with high diversity and cell‐specific production rates in the oceans (Rii et al., 2016; Vaulot et al., 2008). So far, with the rapid development of DNA sequencing technology and in particular, the application of high‐throughput sequencing (HTS) combined with metabarcoding approaches, studies suggested that the high diversity of NFs and PPEs belonging to several divisions in terms of taxonomy (Guo et al., 2023; Moon‐van der Staay et al., 2001). This warrants further studies to explore the hidden world of these communities, especially the distribution of the dominant organisms and their significance to the microbial food web, and thus the entire ecosystem (Giner et al., 2016; Zhu et al., 2005).

At present, fluorescent in situ hybridization (FISH), real‐time quantitative polymerase chain reaction (qPCR) and HTS are commonly used to detect and quantify the microbial community. FISH conducts absolute quantitative research on the specific taxa group at the cellular level (Beisner et al., 2019), while the latter two carry out absolute and relative quantification at the rDNA molecular level (Bonk et al., 2018). The common principle of these three methods is using specific primers or oligonucleotide probes to match with the conserved regions of rDNA of the specific taxa groups. However, qPCR technology could only quantify the rRNA molecular copy number of a specific taxa group but not the cell abundance itself (Botes et al., 2013), while the method of HTS is often queried due to the various rRNA molecular copy numbers of different taxa when it detects the relative abundance of each taxa in the whole community (Keeling & del Campo, 2017). FISH, on the other hand, is thought to be the most straightforward quantitative monitoring technique that could directly detect the cell number of a specific group with a fluorescence microscopic view, thus often used in cell counting and feeding experiments (Beisner et al., 2019; Li et al., 2022). However, the limitation of FISH is subject to the time‐consuming and low degree of automation, compared to the qPCR and HTS which could detect the microbial community from a large sampling size quickly and thoroughly. Compared with the conventional FISH technology, FISH with Tyramide Signal Amplification (TSA‐FISH) also known as CAtalyzed Reporter Deposition FISH (CARD‐FISH), has higher sensitivity and a wider detection range of the cellular RNA molecular signal. In this method, the oligonucleotide probe linked with a horseradish peroxidase (HRP) enzyme is specifically bound to the target sequence, which could catalyse the fluorophore‐labelled tyramide to produce a large amount of biotin permanent deposition in the probe hybridization environment and thus to amplify the fluorescence detection signal geometrically (Riou et al., 2017). Therefore, the low number of RNA molecules in microbial cells could be detected after being hybridized with a probe and bonded with fluorescence (Pernthaler et al., 2002). The detection limits of TSA‐FISH can be as low as 7.4 16S rRNA molecules per cell, indicating 26–41 fold higher sensitivity of TSA‐FISH than that of conventional FISH (Hoshino et al., 2008). Using TSA‐FISH, prevailing distributions of several pico‐ or nano‐eukaryotic groups, that is, MALVs, Chlorophyta, Prymnesiophyceae, MAST, Cryptophytes, Haptophytes and Chrysophyceae have been discovered in many marine areas, accounting for the major proportions of picoeukaryotes and NFs communities (Giner et al., 2016; Lin et al., 2017; Massana et al., 2006; Piwosz & Pernthaler, 2010; Unrein et al., 2014; Wu et al., 2017). Overall, TSA‐FISH is highly recommended for the microscopic detection of specific marine picoplankton when the experimental conditions permit.

*Micromonas* is one of the most common genera of green alga Chlorophyta (Chlorophyta, Mamiellophyceae, Mamiellales, Mamiellaceae, *Micromonas*), characterized by its unicellular pear‐shaped body (cell size 1–3 μm) with a single flagellum and pigment signatures (Simon et al., 2017). Thus, *Micromonas* has the potential to be the dominant contributor to PPE and PNF communities in the ocean (Sherr et al., 2003). Previously, the approach of HTS was mainly used to explore the biodiversity, community composition and biogeographic distribution of natural assemblages of microbial eukaryotes in many studies, where the relative abundance of *Micromonas* and its biogeography was also realized (Guo et al., 2020; Marquardt et al., 2016; Massana et al., 2015). However, the relative abundance of *Micromonas* and its role in the microbial community could be rather underestimated with the HTS approach, because its genomic information could be covered by the high number of rRNA gene copies of the larger protists with larger genomes (Keeling & del Campo, 2017). Thus, the absolute quantification of the cell number of this small eukaryote is highly needed to reveal its abundance more precisely. So far, only a few studies have been conducted to quantify the abundance of *Micromonas* with the FISH method, which demonstrated that *Micromonas* was widely distributed in different marine ecosystems. These studies ranged from the equatorial waters to temperate coastal waters as well as polar oceanic waters, for example, the Arctic seas and the Indian Ocean (Foulon et al., 2008), the English Channel (Not et al., 2004), the Mediterranean Sea (Zhu et al., 2005) and other European coastal area (Giner et al., 2016). However, few quantitative researches on the distribution of *Micromonas* have been carried out in the Northwest Pacific Ocean as well as its marginal seas (Wu et al., 2017). In addition, whether the pigmented *Micromonas* are mixotrophy is still controversial according to the current research. Evidence of phago‐mixotrophy in *Micromonas* and its significant impact on the prokaryotic population has been previously obtained in laboratory and field experiments in many studies (McKie‐Krisberg & Sanders, 2014; Sanders & Gast, 2012; Vaulot et al., 2008). However, other studies also found no clear phagocytosis behaviours in *Micromonas* (Edwards et al., 2023; Jimenez et al., 2021). The inconsistency may be caused by the adaptive evolution of different species or clades and the dynamic trade‐offs related to environmental factors and food conditions that constrain mixotrophic metabolisms (Mansour & Anestis, 2021; Millette et al., 2023). Therefore, the flexible trophic strategies of pigmented *Micromonas* can to some degree be inferred by the relationship between its assembly and certain environmental conditions.

In this study, the first aim was to explore the distribution of *Micromonas* and its roles in the NFs and PPE assemblages in the northern South China Sea (SCS). We performed TSA‐FISH to specifically detect and quantify the cell abundance of *Micromonas* across three water layers along the three transects extending from coastal waters (nearshore area) to oceanic waters (offshore area) and in one diel‐continuous‐observation station in the northern SCS. Furthermore, the environmental abiotic and biotic factors and spatial factors were measured to assess the potential ecological effects on the distribution of *Micromonas*. We hypothesized that (i) referring to the prevailing distribution of *Micromonas* in world's other oceans, we speculated that it was also widely distributed in the marginal sea of the Northwest Pacific Ocean with specific vertical and horizontal spatial distribution patterns and was dominant in PPE and NFs communities; (ii) except the facilitation from the photosynthesis, the dominance and prevalence of *Micromonas* may benefit from its potential mixotrophic life strategy that could become a survival advantage in the changing environmental conditions. This can be inferred from the important role of biotic factors (i.e. prey–predator interactions between *Micromonas* and bacterioplankton) in *Micromonas* assembly; (iii) *Micromonas* assemblages were affected by dispersal limitation due to the large spatial scale of the studied area. These predictions provide an indirect assessment of the vital role of the flexible resource acquisition strategies of *Micromonas* in shaping its ecological niche in the microbial food web, which makes it widely adapt to the environment from the surface to the mesopelagic zone across the northern SCS.

## EXPERIMENTAL PROCEDURES

### Field sampling and processing

**FIGURE 1:**
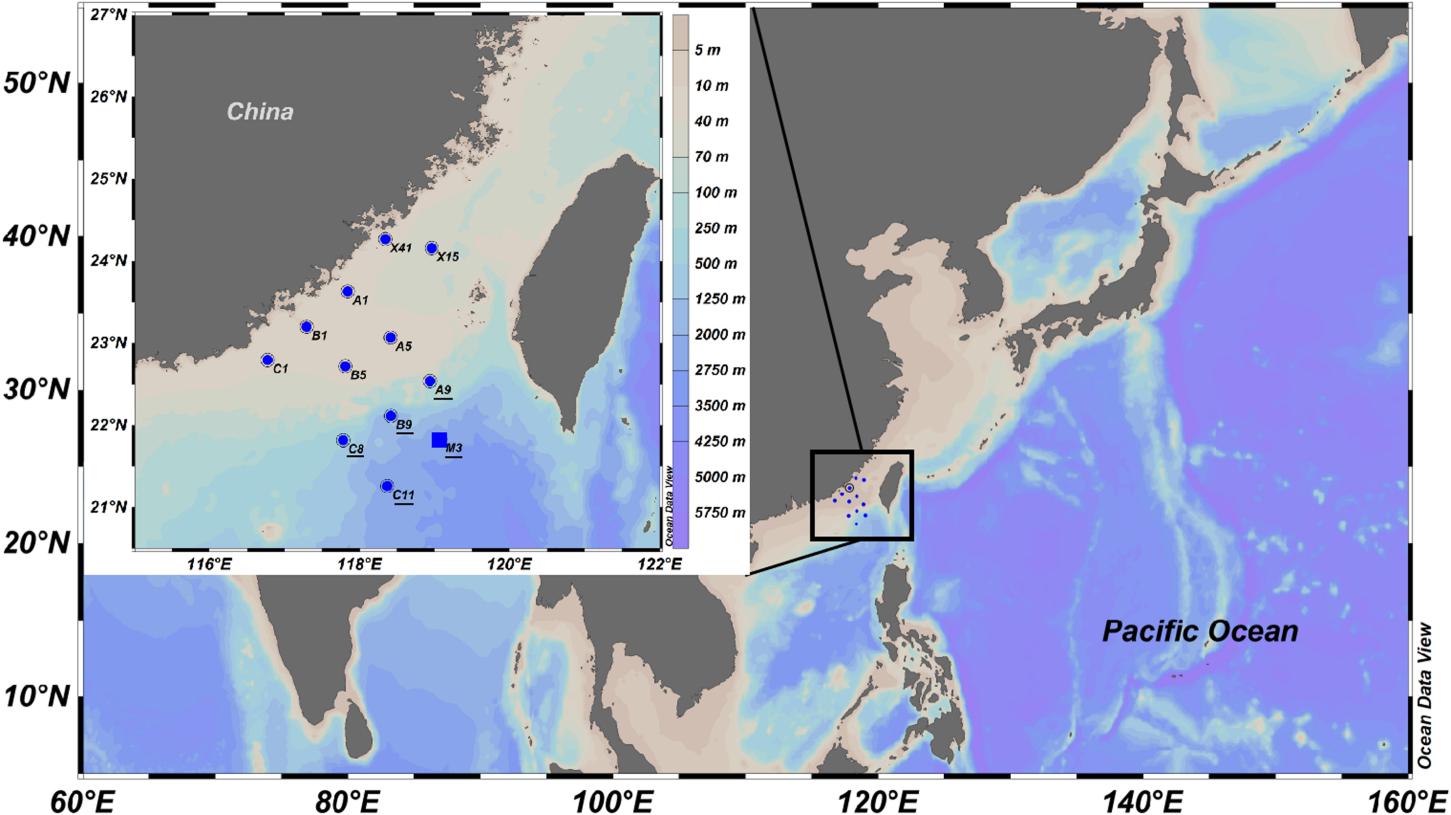
Sampling stations in the northern South China Sea during summer. The station labelled with a solid square was the diel continuous observation station, M3. Sample labels with underlines indicate offshore stations (deeper than 75 m), whereas others represent nearshore stations (lower than 75 m). Maps of sampling stations were made by Ocean Data View 5 software (Schlitzer 2011).

Samples were taken from 11 large‐scale‐observation stations along the three transects extending from coastal waters to oceanic waters and one diel‐continuous‐observation station M3, onboard R/V ‘Yanping II’ in the northern SCS in the summer of 2018 (Figure 1; Table S1). Water samples were collected from the surface layer, deep chlorophyll maximum (DCM) layer and bottom layer in each large‐scale observation station. Samples at the diel‐continuous‐observation station, M3, were collected from 2, 25, 50, 75 (DCM), 100, 200 and 500 m water depths in daytime and night, respectively. Water samples were collected by a Seabird 19 CTD (SBE917plus; SeaBird, Bellevue, WA). In this study, the 1000 m water layer was recognized as the bottom layer in the basin stations (B9 and C11) because of the limitation of CTD sampling depth. Stations with a depth of less than 75 m (Station A1, A5, B1, B5, C1, X4 and X15) were considered the nearshore station, while others were recognized as the offshore station (Station C8, C11, A9, B9 and M3).

In this study, the environmental factors included abiotic parameters (i.e. temperature, salinity, depth, dissolved inorganic nitrogen [DIN] and dissolved inorganic phosphorus [DIP]) and biotic parameters (i.e. chlorophyll *a* [Chl *a*], cell abundance of bacteria, *Synechococcus*, PPEs, NFs and ciliates) of the water sample were measured to describe the environmental conditions of the studied area and thus to assess the potential effect of the environmental factors on the target organism. Technical details of the physicochemical and biological analyses were described in Supporting Information Text S1.

### Testing probe in silico specificity and its optimal hybridization condition

Due to continuous improvement of the biological database and long‐time utilization of the oligonucleotide probe, it is necessary to re‐evaluate the in silico specificity of probes and retest their optimal hybridization conditions which are mainly affected by the concentration of formamide, a key ingredient in hybridization buffer (Riou et al., 2017). The proper concentration of formamide can facilitate distinguishing the target and non‐target taxa (e.g. false‐positive control) effectively and reduce the interference of non‐target cells (Daims et al., 1999).

Probe Micro 01 (5′‐AAT GGA ACA CCG CCG GCG‐3′) was designed to detect the genus *Micromonas* according to Not et al. (2004). So far, there are four species affiliated to this genus, that is, *M*. *pussila*, *M*. *commode*, *M*. *bravo* and *M*. *polaris*. However, before the discovery of *M*. *commode*, *M*. *pussila* was recognized as the only species in this genus (Simon et al., 2017). Thus, to re‐evaluate the specificity of Micro 01, the sequence of the probe Micro 01 was input into the SILVA ‘SSU Ref’ database under ‘TestProbe’ (http://www.arb-silva.de/search/testprobe/) to contrast with all described sequences, following the TestProbe tutorial (https://www.arb‐silva.de/documentation/testprobe‐tutorial/). SILVA rRNA gene database is identified to be the most comprehensive, up‐to‐date and quality‐controlled bioinformatics database which contains aligned rRNA gene sequences from the Bacteria, Archaea and Eukaryota domains, whereas the target group coverage of rRNA gene‐targeting probes could be tested in silico using the probe match and evaluation tool (Quast et al., 2013). A maximum number of central mismatches was installed at three, and the matching results under different central mismatches (0, 1, 2 and 3) were performed in turn in ‘Browser’. Then, an outgroup (non‐targeted) cultured strain (*Chlorella sphaerica*, SAG11.88), with its rRNA sequence being the fewest central mismatch and the closest with the targeted sequence, was selected as the false‐positive control for the target taxa, abbreviated as (−)pCtrl and used to validate the probe specificity and ensure the best hybridization condition (Table S2). A cultured strain (*M*. *pussilla*, CCMP1545) belonging to the group of interest targeted by the probe was selected as the positive probe control abbreviated as (+)pCtrl (Figure S1; Table S2). Both cultured cells were harvested in the early stationary growth phase and fixed for 1 h at room temperature with 1% buffered paraformaldehyde (PFA, w: vol) final concentration, and clumps of cells were vortexed to disaggregate. After this step, the fixed cells were quickly frozen at −80°C until further processing.

FISH with HRP‐labelled probe associated with tyramide signal application (TSA‐FISH) was performed generally referring to Not et al. (2002), Riou et al. (2017), Wu et al. (2017) and FISH protocols in SILVA (https://www.arb‐silva.de/fish‐probes/fish‐protocols/). The series of TSA‐FISH steps are briefly mentioned below, and when detailed procedure is provided in Supporting Information Text S2. Fixed cells were embedded and dehydrated to minimize cell loss. Enzymatic permeabilization treatment for the penetration of the HRP‐probe was no need for picoeukaryotes because they lack cell walls. Hybridization with an HRP‐coupled oligonucleotide probe (Table S2) was conducted in the dark and followed by a washing step. The stringency of the hybridization conditions was optimized by adjusting the concentrations of formamide (30%, 35%, 40%, 45% and 50%) in the hybridization buffer and NaCl in the washing buffer (Supporting Information Table S2). Probe hybridization was then revealed by a TSA reaction using 1× Alexa Fluor™ 488 (green fluorescence) labelled Tymamide Reagent (Thermo Fisher Scientific Inc., Germany) and cellular DNA was DAPI‐stained (blue fluorescence), before mounting with glycerol medium on the slide and being identified with epifluorescence microscopy (Leica DM 4500B).

### TSA‐FISH on environmental samples

**FIGURE 2:**
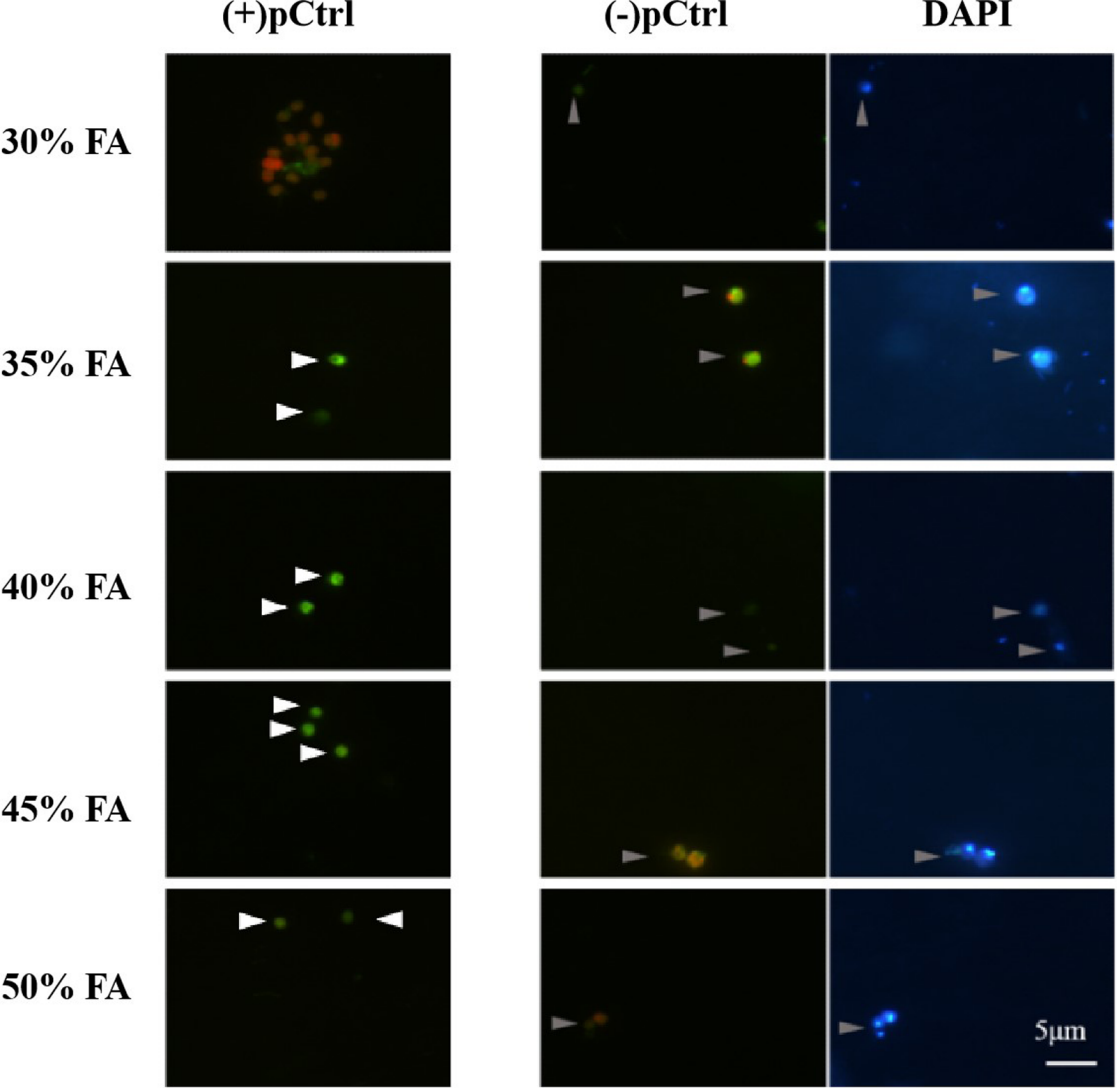
Hybridization performances of Micro 01 in different formamide (FA) concentrations. Strains of the (+)pCtrl and (−)pCtrl are shown in Supplementary Table S2. Cells dyed with Alexa Fluor 488TM and DAPI are supposed to perform bright green and blue under the blue and UV excitation light of the epifluorescence microscopy, respectively. The white arrows point at the positive control. The grey arrows point at unspecific labelled cells, that is, false‐positive control. Scale bars indicate 5 μm.

The seawater samples of 450 mL were pre‐filtered through 20 μm nylon mesh and 3 μm polycarbonate membranes successively and then were immediately fixed in 50 mL of 10% PBS‐buffered PFA (1% final concentration, Sinopharm Chemical Reagent Co., Ltd, China) for 1 h at room temperature. The fixed samples were then filtered onto 0.8 μm polycarbonate membranes under 200 mmHg pressure and dehydrated in an ethanol series (50%, 80% and 100%, 3 min each), which were finally stored at −80°C until analysis. A 2‐μm‐pore‐size membrane was put under the 0.8 μm polycarbonate membrane to confirm filtration uniformity. The cell numbers of *Micromonas* were determined using the HRP‐labelled probe Micro 01 under the above‐described procedure of TSA‐FISH, with the optimal formamide concentration of 40% in hybridization buffer as the result shown below (Figure 2; Figure S2).

### Statistical analysis

The significance of the spatial difference in the distribution of *Micromonas*, PPE and NFs, as well as the proportions of *Micromonas* in its related microbial communities (PPE, PNFs and NFs communities) was analysed using one‐way ANOVA, followed by Duncan means comparisons on samples grouped by water layers (surface, DCM and bottom) and habitats (nearshore & offshore). The analyses were carried out using the statistical program SPSS 22 and data were reported as averages and standard deviation (SD) and statistical difference was accepted at *p* < 0.05.

To analyse the impact of environmental factors on the spatial distribution of *Micromonas*, principal component analysis (PCA) was first used to test the explanatory power of environmental variables on the variation of the samples. Correlation analyses based on Pearson's rank correlation coefficients were conducted to assess the relationships among the environmental parameters (including abiotic and biotic factors) with the abundance of *Micromonas*. The Bray–Curtis distance among samples based on *Micromonas* abundance was further correlated to the Euclidean distance among samples based on each environment variable by Mantel tests. We also conducted a univariate linear model (LM) with the Ordinary Least Square method to regress the abundance of *Micromonas* and environmental variables, aiming to identify the variables that have significant effects. First, environmental factors with a high variance inflation factor (VIF > 20) were eliminated to avoid collinearity. Then, we performed two methods to do the model selection to establish the best linear model for the quantitative relationship between *Micromonas* abundance and variable factors. The first method was backward selection, where we first included all available variables in the LMs and then step‐wisely removed insignificant variables based on their *p* values and Akaike Information Criterion (AIC) weights. The other one applied a multimodel inference procedure using the function dredge in the R package MuMIn, first creating a set of models with all possible combinations of the initial variables, and then ranking them by AICc fitted with Maximum Likelihood. A subset of models with ΔAICc <2 were selected and their parameters and associated *p* values were estimated using the model averaging approach. Finally, the best model was selected by function sw(), keeping variables with the value of the sum of weights >0.5 (Gross et al., 2017). In addition, spatial variables of each sampling site were generated to account for the spatial autocorrelations among stations, using the principal coordinates of neighbour matrices (PCNMs) approach based on the longitude and latitude coordinates (Dray et al., 2006). These spatial eigenvectors were always included in the backward selection processes to account for the effects of dispersal because microbial organisms are passive dispersers (Hanson et al., 2012). Furthermore, the relative contributions of the variations of environmental and spatial factors to *Micromonas* assemblages (represented by their cell abundance) were explored using variance partitioning analysis (VPA) with adjusted *R* ^2^ coefficients based on redundancy analysis (RDA; Legendre, 2008). Before VPA, factors with VIF > 20 were also eliminated, followed by the forward selection procedure to screen the significant environmental and spatial variables (Blanchet et al., 2008). All environmental factors and abundance data were log(*x* + 1) transformed before analyses. All these statistics analyses were performed in R (v.4.3.0) unless stated otherwise.

## RESULTS

### Specificity of probe Micro 01

Search results for the generated proof against the SILVA SSU Ref database showed that there was a total of 368 sequences affiliated with *Micromonas* (Table S3). Probe Micro 01 had complete specificity with zero central mismatch, with 112 sequences affiliated to *Micromonas*, accounting for 30.43% of the total target sequences. When the number of central mismatches was 1, there were 350 sequences belonging to *Micromonas*, accounting for 95.11% of the number of total target sequences. The non‐target sequences mostly belonged to other genera of Chlorophyta. When the number of central mismatches came to 2, the total matched sequences increased largely to 3795, while only eight of the matched target sequences were newly added. No new matched target sequence was found with three central mismatches, although the total matched sequence increased nearly four times (16,667) than with two central mismatches. Overall, when the allowable central mismatch number was 3, the oligonucleotide probe Micro 01 had 97.28% coverage of the total target sequences of *Micromonas*, with only 10 target sequences unmatched (Table S4).

### Optimal formamide concentration

Test of the optimum formamide concentration of Micro 01 showed that Micro 01 efficiently hybridized to both positive control and false‐positive control at 35% and 45% formamide concentrations (Figure 2), indicating that they were indistinguishable in the same field of vision under these conditions. When the formamide concentration was at 30% or 50%, the fluorescence intensity of the positive control was relatively low and hard to identify. However, the positive control showed a much brighter green than the false‐positive control under the formamide concentration of 40% (Figure 2), thus allowing clear identification of the target cell and distinction from the false‐positive control, which indicates an optimal formamide concentration of 40% to achieve best hybridization effect.

### Distribution patterns of *Micromonas* in the northern SCS

**FIGURE 3:**
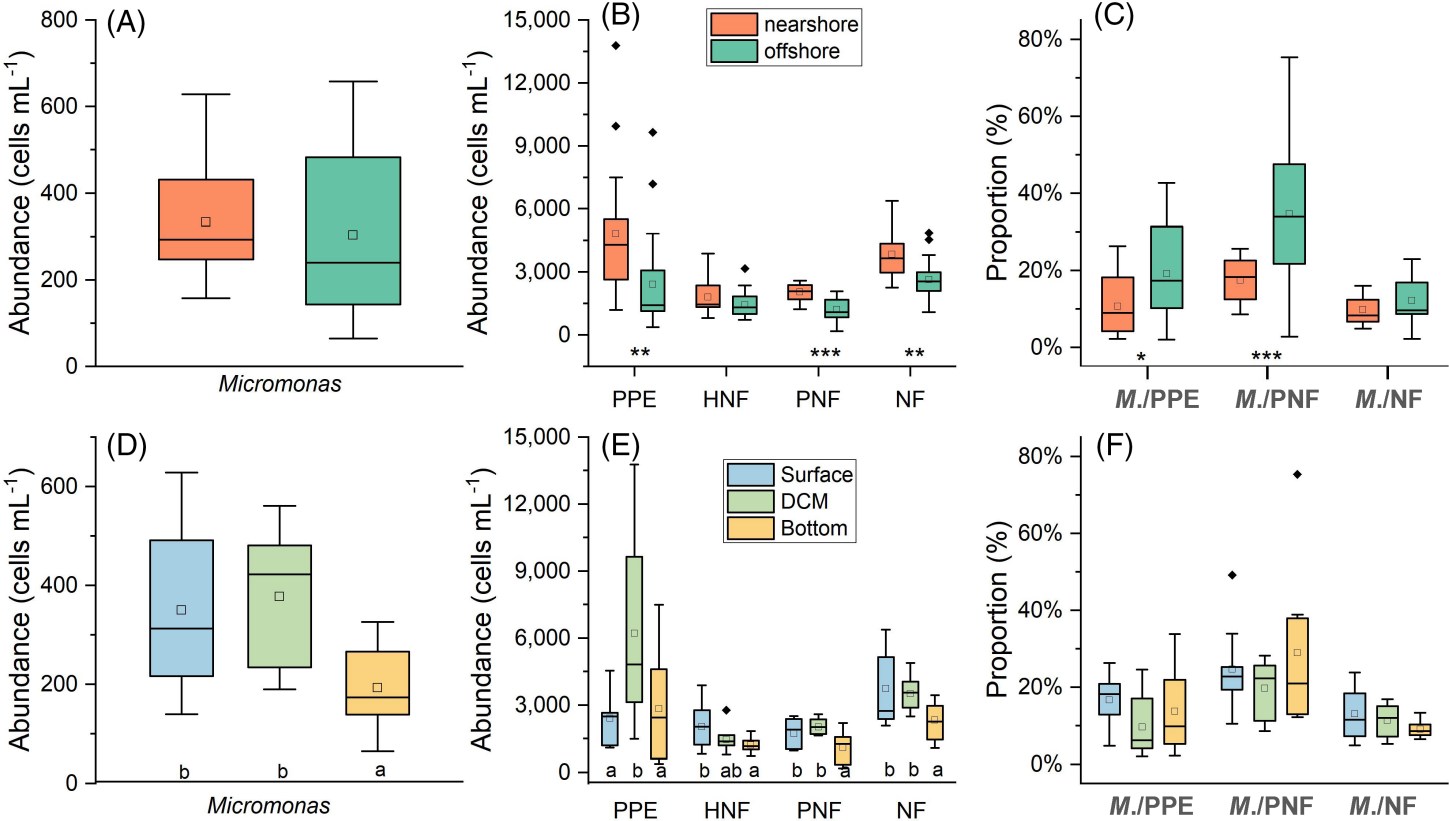
Boxplots displayed the spatial distribution of the abundance of *Micromonas*, PPE and NFs (a, b, d, e), as well as the proportions of *Micromonas* in PPE, PNFs and NFs communities (c, f) in the northern South China Sea. One‐way ANOVA and Duncan's means comparisons were used to compare the differences among different groups (layers and habitats). Asterisk indicates significant differences within the groups of habitats at the level of *p* < 0.05 (*p < 0.05; **p < 0.01; ***p < 0.001). Different letters below the box indicate significant differences within the groups of layers at the level of *p* < 0.05. No asterisk or no letter indicates no significant difference within the groups. The hollow diamonds represent the average values of the individual index in each group. *M*. represents the abbreviations of *Micromonas*. Variable abbreviations are as follows: HNF, heterotrophic nanoflagellate; *M*. represents the abbreviations of *Micromonas*; NF, total nanoflagellate including HNF and PNF; PNF, pigmented nanoflagellate; PPE, photosynthetic picoeukaryotes.

*Micromonas* in all environmental samples were detected using the TSA‐FISH with the optimal formamide concentration in hybridization buffer at 40%. The results showed that *Micromonas* abundance displayed higher abundance in the nearshore (333 ± 130 cells mL^−1^) than offshore area (303 ± 174 cells mL^−1^), although the difference was not significant (Figure 3A; Table 2). The abundance of *Micromonas* was slightly higher in the DCM (377 ± 130 cells mL^−1^) than in the surface (350 ± 156 cells mL^−1^), while both were significantly greater than the abundance in the bottom (193 ± 83 cells mL^−1^; Figure 3D; Table 2). However, the horizontal distributions of *Micromonas* in three water layers and vertical distributions along the three transects from nearshore to the open ocean did not show some regular trends (Figure S3a–c and S4a–c). In addition, *Micromonas* abundance seemed to display more strengthened stratification from nearshore to offshore from the view of sections, probably due to the largely increasing depth (Figure S4a–c).

**FIGURE 4:**
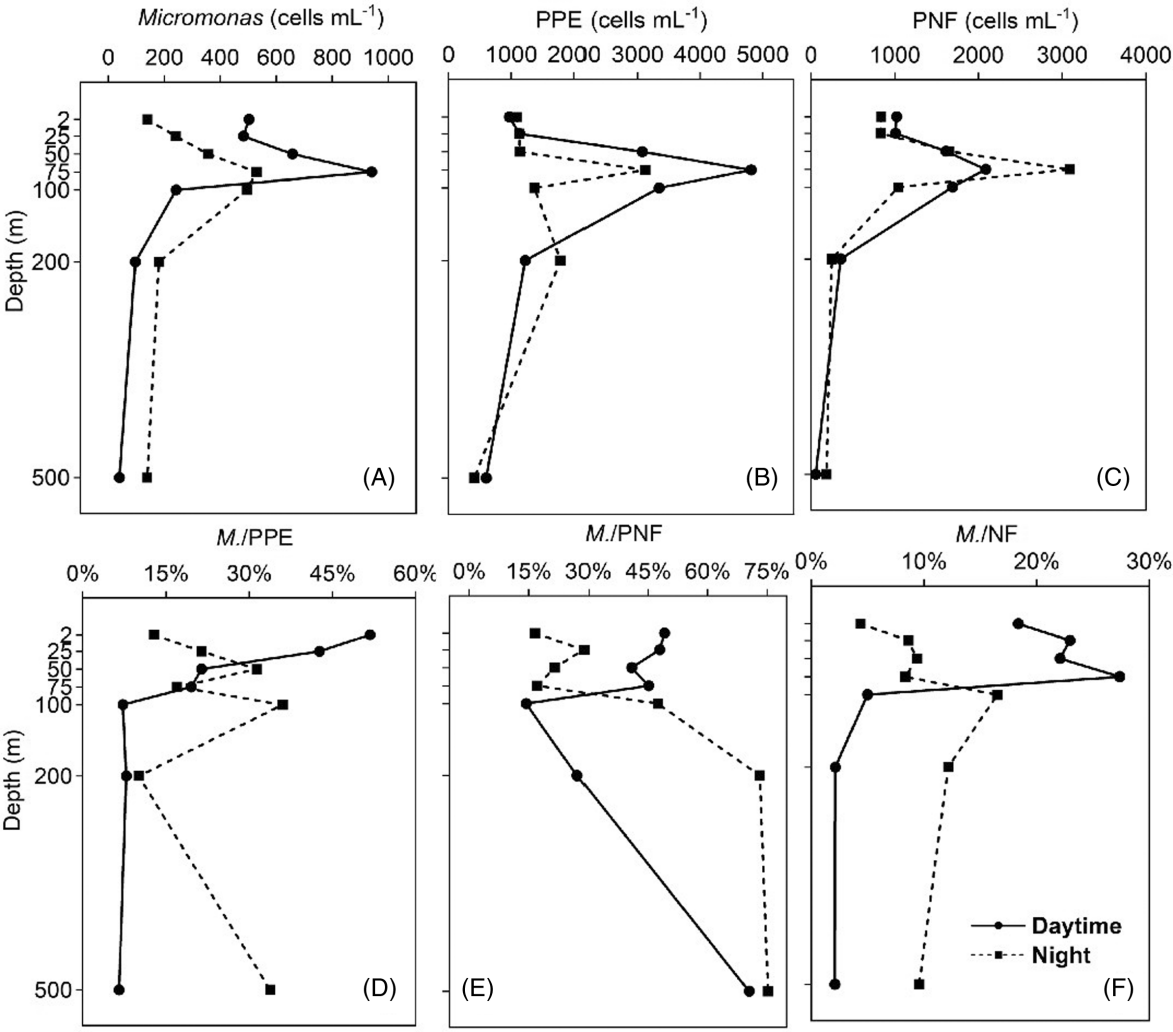
Diel vertical distributions of the abundance of *Micromonas* (A), PPE (B) and PNFs (C), as well as the proportions of *Micromonas* in PPE (D), PNFs (E) and NFs communities (F) in M3 station. The depth of the DCM layer was in the 75 m for both daytime and night. Refer to Figure 3 for variable abbreviations.

Results from the diel continuous observation station (M3) showed a general vertical distribution trend of the abundance of *Micromonas*, PPE and PNFs communities, which was increasing first to the DCM layer (75 m) or 100 m and then decreasing with the increase of water depth (Figure 4A–F). The abundance of *Micromonas* reached the maximum values in the DCM layer, which were 940 and 530 cells mL^−1^ in the daytime and at night, respectively (Figure 4A). As for the diurnal difference, it appeared that *Micromonas* abundance displayed a larger vertical variation during daytime, with higher abundance in the upper part of the epipelagic zone (depth < 100 m) and lower abundance in the deep layers (100, 200 and 500 m) in the daytime than that at night (Figure 4A), probably due to the higher irradiance exposures in the upper layer during daytime in summer that facilitate the growth of the photosynthetic organisms.

### Contribution of *Micromonas* in the PPE and NF communities

For the community of PPE, the abundance in the nearshore area was significantly higher than the offshore area (Figure 3B), with the highest in these two areas both occurring in the DCM layer (Table 2), leading to significantly lower abundance in the surface and bottom layer (Figure 3E). However, the proportion of *Micromonas* in the PPE community seemed to display the opposite trends in different layers and habitats compared with their abundance distribution. Specifically, the ratio of *Micromonas* to PPE was higher in the offshore (19.14%) than the nearshore area (10.65%) and higher in the surface (16.66%) and bottom layer (13.67%) than the DCM (9.57%; Figure 3C,F; Table 2). In the M3 station, the proportion of *Micromonas* in the PPE community decreased with the increase of the water depth in the daytime, with the maximum value being 51.7% in the surface layer and dropping largely to less than 8% in the deep depths (100, 200 and 500 m; Figure 4D). The proportion at night, however, fluctuated greatly along the depth, with the high‐value inflection points appearing at 50 m (31.32%), 100 m (36.01%) and 500 m (33.72%) (Figure 4D). Overall, an average proportion of 15.39% indicated the dominance of *Micromonas* in the PPE community in the studied area.

To figure out the contribution of *Micromonas* in the NFs community, which consists of both the HNFs and PNFs sub‐communities, the abundance of HNFs and PNFs and their proportions in the whole NFs community were also compared. Similarly, the abundance of HNFs, PNFs and total NFs was higher in the nearshore than in the offshore area, as well as higher in surface and DCM layers than the bottom layer (Figure 3B,E; Table 2). PNFs displayed higher proportions in NFs in the DCM layer across the whole area and bottom layer of the nearshore area, which were at the depth < 100 m above the euphotic zone, while HNFs mostly dominated in the surface layer (3 m) and deep layers (100, 200, 500 and 1000 m; Table 2). As an important component of bacterivorous photosynthetic flagellates, the proportions of *Micromonas* in the PNFs sub‐community displayed the opposite trend in different layers and habitats compared with their abundance distribution. Specifically, the ratio of *Micromonas* to PNFs was significantly higher in the offshore (34.58%) than in the nearshore area (17.41%) and higher in the bottom layer (28.87%) than the surface (24.60%) and DCM (19.70%; Figure 3C,F; Table 2). In M3 station, *Micromonas* composed more than 40% PNFs in the upper part of epipelagic zone (depth < 100 m) during the daytime, while dropped largely in the 100 m (14.35%) and 200 m (2.72%; Figure 4E). However, there was an opposite trend at night that the average proportion in the upper part of epipelagic zone (21.09%) was much lower than that in the deeper layers (Figure 4E). It was interesting that *Micromonas* displayed a rather large proportion in the PNFs at 500 m depth whether in the daytime (70.49%) or at night (75.30%; Figure 4E). Similar pattern was observed in the proportion of *Micromonas* in the total NFs community, except at 500 m depth (Figure 4F). It was the obvious advantages of the relative abundance of HNFs to PNFs in the deepest layer (500 m) that led to the incredibly large proportion of *Micromonas* in the PNFs but rather low proportion in the whole NFs community in this depth (Figure S5). Overall, the average proportions of 26.73% and 10.94% of *Micromonas* in the PNFs sub‐community and total NFs community indicated its significant contribution to the NFs communities in the studied area.

### Correlation of *Micromonas* abundance with the environmental and spatial factors

From the ordination biplots of PCA, the environmental characteristics in the sampling area displayed apparent spatial differences for both the large‐scale‐observation stations and diel‐continuous‐observation station M3 whether accounting for abiotic, biotic factors, or both into the analyses (Figure S6). The difference among water layers was subject to sampling depth and salinity, with deeper layer corresponding to higher salinity (Figure S6A,E), while the difference between habitats was mainly subject to biotic factors, with the nearshore habitat having more abundant microbial organisms than offshore habitat (Figure S6C,E). For the diel‐continuous‐observation station M3, the environmental difference was mainly reflected among different water layers, with a weak difference between daytime and night (Figure S6B,D,F). Samples in the mesopelagic zone in the M3 station were characterized by high salinity, depth, DIN and DIP, while samples above the epipelagic zone generally with high values of temperature and abundance of bacteria, PNF and PPE (Figure S6f). For all the samples, the environmental abiotic and biotic factors, the abundance of *Micromonas* and the proportions of *Micromonas* in its related microbial communities showed vertically distributed patterns with the increase of sampling depth, although some linear relationships were not significant (Figure S7A–L).

**FIGURE 5:**
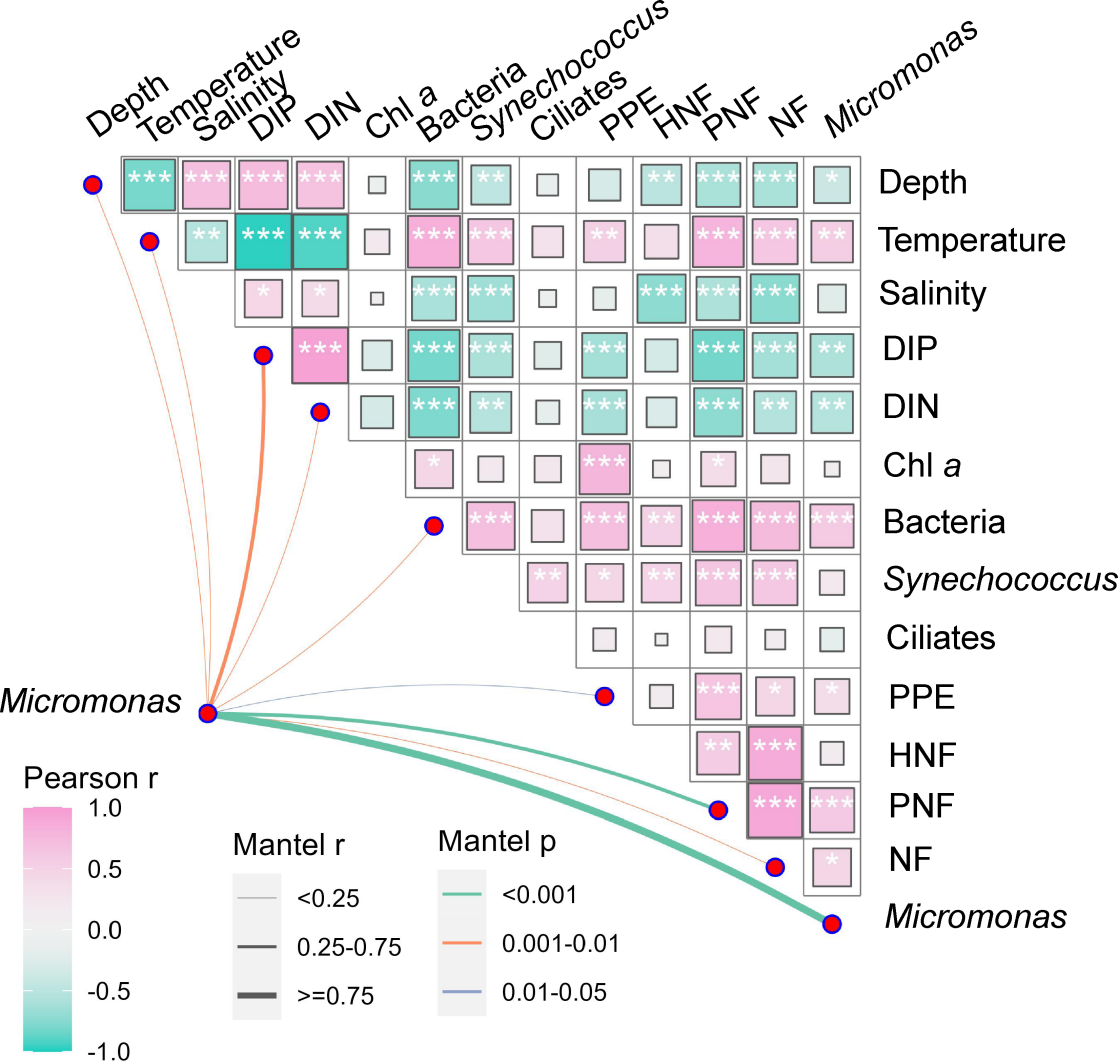
Environmental drivers (including abiotic and biotic factors) of the abundance of *Micromonas* in the northern South China Sea. Pairwise comparisons of environmental variables are shown with a colour gradient denoting Pearson's correlation coefficient. The Bray–Curtis distance based on the abundance of *Micromonas* was related to each environment variable (Euclidean distance) by Mantel tests. All the variables are log(*x* + 1) transformed. Significant relationships labelled with asterisk (*p < 0.05; **p < 0.01; ***p < 0.001) based on 9999 permutations were shown. Refer to Figure 3 for variable abbreviations.

**FIGURE 6:**
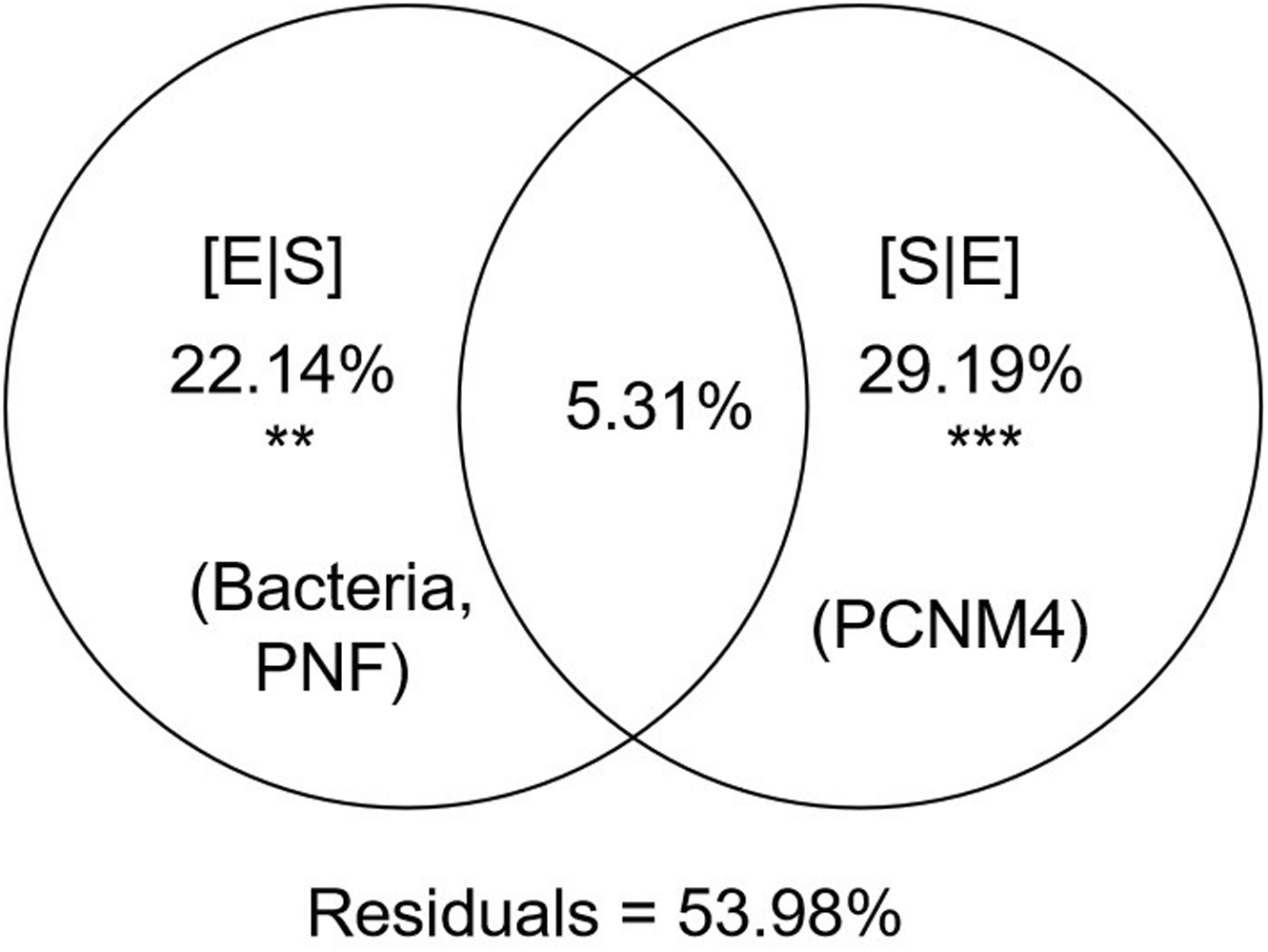
Variation partitioning analyses (VPA) for the *Micromonas* assemblages for all studied samples. Pure environmental [E|S] and pure spatial [S|E] components represent the relative importance of environmental filtering (i.e. bacteria and PNF) and spatial factors (i.e. PCNM4), respectively, after screening the variables with low variance inflation factor (VIF < 20) and significant correlation (*p* < 0.01) with *Micromonas* abundance. The shared fractions are provided. Values are statistically significant based on 999 permutations (**p < 0.01; ***p < 0.001). The residual proportion represents the unexplained variance.

To figure out the relationship of *Micromonas* abundance with the environmental factors, Pearson's correlation analysis (two‐tailed test, *p* < 0.05) was first conducted on all samples. Results showed that the abundance of *Micromonas* was positively correlated with temperature and negatively affected by water depth, DIP and DIN (Figure 5). For the biotic factors, *Micromonas* had positive relationship with the abundance of bacteria, PPE, PNF and NF (Figure 5). Similar results were displayed in the univariate linear regression models of *Micromonas* abundance and these factors (Figure S8A–L). In addition, significant relationships between *Micromonas* assembly and water depth, temperature, nutrients, bacterioplankton and NFs were also validated in Mantel tests (Figure 5). Considering the dispersal effect on the passive dispersed microbial organisms, we further test the relative contributions of the environmental and spatial variations on *Micromonas* assemblages. The result of VPA showed that *Micromonas* abundance was significantly explained by the purely environmental (22.14%; *p* < 0.01; represented by bacteria and PNF) and spatial (29.19%; *p* < 0.001; represented by PCNM4) components, after filtering the collinearity and insignificant factors (Figure 6). The best linear model came to the same result as the two model selection methods: *Micromonas* abundance was affected significantly by the spatial variable PCNM4 and several biotic factors, such as PNF, Chl *a*, HNF, bacteria and *Synechococcus* (Tables 1 and S5). These results suggested that the dispersal limitation and biotic factors may play a more important role in *Micromonas* assemblages than abiotic factors in the studied area.

**TABLE 1:** Results of model selection using a multimodel inference procedure with the function dredge in the R package MuMIn.

Variables	Model 1	Model 2	Model 3	Model 4	Model 5	Model 6	N containing models	Sum of weights
HNF	1	1	1	1	1	1	6	1
PCNM4	1	1	1	1	1	1	6	1
PNF	1	1	1	1	1	1	6	1
Chl *a*	1	1	1	1	1	0	5	0.9
Bacteria	1	0	0	1	0	1	3	0.53
*Synechococcus*	1	0	0	1	1	0	3	0.52
Ciliates	0	1	0	1	0	1	3	0.43
PPE	0	0	0	0	0	1	1	0.1
*R* ^2^	0.804	0.78	0.76	0.816	0.774	0.793		
adj. *R* ^2^	0.764	0.744	0.729	0.77	0.736	0.75		
AICc	35.2	36	36.1	36.6	37.1	37.2		
ΔAICc	0	0.82	0.87	1.38	1.91	2		
Weight	0.281	0.186	0.182	0.141	0.108	0.103		

*Note*: A total of six best‐selected models with ΔAICc ≤2 were listed. The variables that were included in the model were presented as 1 while being presented as 0 if not. The number of containing models and the sum of the weights of each variable were shown. The *R* ^2^, adj. *R* ^2^, Akaike Information Criterion (AICc), ΔAICc and the weights of each best‐selected model were presented. According to these parameters, Model 1 was selected as the best linear model with variables HNF, PNF, Chl *a*, bacteria, *Synechococcus* and PCNM4 kept in. Refer to Figure 3 for variable abbreviations.

## DISCUSSION

### Evaluating the specificity of the probe in TSA‐FISH

Since there are continuous discoveries of the strains and improvements in the sequence database, it was necessary to re‐evaluate the specificity of the probes before using them for downstream analysis via TSA‐FISH. For the specific probe, sequences not only need to be matched with the target group but also should have at least one central mismatch with the non‐target organisms (Riou et al., 2017). Usually, when the number of central mismatches is more than 3, the matched sequences are considered as the non‐target sequences. Nevertheless, because of the high in situ phylogenetic diversity of the marine organisms and the ambiguous classification status of some strains, it remains challenging to design and ensure a probe with absolute specificity that is capable of covering all target sequences without any mismatch. Even with three central mismatches, it would still be possible that some target sequences failed to match. Hence, what can be done is to make sure that the designed probe has as much specificity as possible. In this study, the oligonucleotide probe Micro 01 has been used for more than 15 years since designed by Not et al. (2004) with a satisfactory specificity condition, matching sequences that accounted for 97.28% (Table S3). When the number of the central mismatch increased from 0 to 1, the matched target sequences increased two times, however, non‐target sequences also appeared. Most of the non‐target sequences belonged to the genus in class Mamiellophyceae, only a few were affiliated with some freshwater organisms under Chlorophyta, and others belonged to Alveolata and Heterokonta that had larger cell sizes than *Micromonas* (Vaulot et al., 2008; Zhu et al., 2005). Thus, the influence of these non‐target organisms on the abundance calculation of *Micromonas* can be greatly reduced through filtering in situ in this study.

However, not only the presence of new target strains can affect the hybridization efficiency of the probe, but also the non‐target organisms with similar phylogenetic or sequence composition to the target group have a great impact on hybridization results. In addition to comparing the probe against the sequences in the database, optimization of the hybridization condition that is mainly affected by the formamide concentration is also necessary. This step can help effectively distinguish the target and non‐target groups and reduce the influence of the false‐positive detection of outgroup on the hybridization results (Daims et al., 1999). The concentration of the formamide applied in the previous studies was 40% (Not et al., 2002, 2004), similar to our result (Figure 2). Overall, our results suggest the optimum formamide concentration of 40% for high specificity of oligonucleotide probe Micro 01.

### The advantages and bias of TSA‐FISH in quantifying specific species group

As a precise indication technique used to detect and quantify specific taxa groups within the microbial community in natural samples, the major advantage of the conventional FISH or TSA‐FISH is that it counts cell numbers directly without thinking about the rDNA genes that usually occur in multiple copies and of which the copy number varies greatly among different taxa, compared with other PCR‐based approaches (e.g. qPCR, HTS technology). This is because the rDNA copy number appears to be correlated to genome size (or DNA content) in eukaryotes (Prokopowich et al., 2003), and thus establishes a good relationship with cell size as demonstrated for phytoplankton (Zhu et al., 2005). Therefore, it could seriously bias estimates of the small cells such as *Micromonas* in the natural samples, due to the strong contribution of a high number of rDNA copies of the larger protists or the gametes of multicellular organisms to the total eukaryotic rDNA pool that would decrease the relative contribution of *Micromonas* (Keeling & del Campo, 2017). However, some reasons probably cause the discrepancy of overestimating or underestimating when observed with FISH. The first is related to the operational process associated with FISH (Not et al., 2002). For example, the target cells may be disrupted or lost during fixation and other treatments such as dehydration in ethanol series or step of washing by detergent. Some taxa may fail to be permeable to the HRP‐labelled probes or can't achieve the best hybridization effect due to suboptimal conditions, and thus may not be labelled efficiently by the probes. The second reason is related to the specificity of the probe, which might not be perfect because it is hard to validate against the outgroups with central mismatches that belong to the uncultured organisms; therefore, some non‐target species may be detected and included in a given estimate (Riou et al., 2017). In conclusion, it should be emphasized that FISH should not be used alone but in conjunction with other molecular biological techniques such as qPCR and HTS, to gain more comprehensive information about the absolute abundance of the specific microbial organisms and their contributions to the whole microbial communities, and thus deeply understand the roles of the specific groups in the entire ecosystem.

### Prevalence and dominance of *Micromonas* in the northern SCS

The specific probe coupled to the TSA‐FISH technique allowed analysis of the distribution pattern of the small flagellate *Micromonas* along environmental gradients in the northern SCS and the role it played in the nano‐ and picoeukaryote assemblages. Previous studies about the distribution of *Micromonas* in the coastal surface waters of the Atlantic Ocean showed high abundance in the Western English Channel (500–7000 cells mL^−1^) and Mediterranean (400–5000 cells mL^−1^) while accounting for an average of 45% and 21% of picoplanktonic eukaryotes in these two areas (Not et al., 2004; Zhu et al., 2005). Studies in Arctic Waters and the subtropical Indian Ocean found that the abundance of *Micromonas* was generally highest near the surface waters and decreased rapidly with depth, ranging from hundreds to thousands of cells per millilitre (Foulon et al., 2008). A decreasing trend from the coastal to the oceanic waters was also observed along a transect across the Indian Ocean (Foulon et al., 2008). In this study, *Micromonas* was detected in all stations sampled, with about 377 cells mL^−1^ in the coastal surface water, significantly larger than the abundance of 116 cells mL^−1^ in the offshore bottom water in the northern SCS (*p* < 0.05, Table 2), generally according with the trends of decreasing with depth and distance from the coast (Figure 3). This range of abundance was considerably lower than the abundance in other oceans reported previously (see above), but was consistent with a study along an estuary‐to‐basin transect in the northern SCS, which showed a maximum abundance of *Micromonas* about 500 cells mL^−1^ in the upper 150 m depth in the coastal waters and lower than 100 cells mL^−1^ in other offshore stations (Wu et al., 2017). However, although the absolute abundance of *Micromonas* was relatively low in the northern SCS, the proportion of *Micromonas* in the PPE community ranged from 1.97% to 42.60% in this study, with the average proportion of 16.5% in the coastal surface water, falling into the range previously observed in the Mediterranean Sea off the Spanish coast (5%–65% over the year with the average of 21%; Zhu et al., 2005). For the vertical distribution, the *Micromonas* counts as well as PPE abundance were the highest mostly at the DCM layer whether in the large‐scale‐observation stations or M3 station in the northern SCS (Figures 3 and 4). Similar patterns were found in the northern SCS, which exhibited a clear subsurface maximum layer for the abundance of *Micromonas* and other PPE groups (Wu et al., 2017). Meanwhile, in upper layer (<100 m), the decreasing trend of the proportions of *Micromonas* in the PPE community showed the opposite result with their increasing abundance (Figure 4A,B,D), which was probably due to a large number of other picoeukaryotes (e.g. Prymnesiophyceae, Pelagophyceae, *Ostreococcus*, *Bathycoccus*) with larger growth rate became more abundant than *Micromonas* in the upper water (Wu et al., 2017). Moreover, we found a range of 2.72%–75.30% (average of 17.41% for nearshore and 34.58% for offshore) for the proportion of *Micromona* in PNF sub‐community and a range of 2.12%–22.95% (average of 9.72% for nearshore and 12.16% for offshore) for the proportion of *Micromona* in total NFs community in this study. This result was comparable with a study about the community composition of microbial flagellates in subtropic–tropic marginal seas of China, which found an average relative abundance of up to 15.04% of *Micromona* in NFs community in the Minzhe coastal waters (Guo et al., 2023). Overall, our results revealed that *Micromonas* was distributed ubiquitously and contributed significantly to the PPE and NFs assemblages in the northern SCS. However, a relatively high abundance (>1000 cells mL^−1^) of NFs was observed unexpectedly at depths of 500 m in station M3 and 1000 m in station B9 (Figure S7J). Previous studies showed that dead microbial cells or cells that are severely stressed metabolically can sink much faster than morphologically similar viable ones (Padisák et al., 2003; Waite et al., 1997). Therefore, the high abundance of NFs in the deep layers might be overestimated due to the sinking of dead cells from the upper layers into deep water that could not be distinguished from live NFs and thus were counted (Huang et al., 2008).

**TABLE 2:** Abundance of *Micromonas*, PPE and NFs (including HNFs and PNFs) and the proportions of *Micromonas* in its related microbial communities in different layers of the nearshore and offshore areas.

	Nearshore			Offshore		
	Surface	DCM	Bottom	Surface	DCM	Bottom
*Micromonas* (cells mL^−1^)	377 ± 156	379 ± 123	244 ± 61	317 ± 168	375 ± 153	116 ± 36
PPE (cells mL^−1^)	2943 ± 1250	6800 ± 4410	4672 ± 1945	1756 ± 751	5505 ± 2890	984 ± 777
HNFs (cells mL^−1^)	2559 ± 1128	1641 ± 527	1265 ± 212	1504 ± 572	1118 ± 307	1138 ± 440
PNFs (cells mL^−1^)	2228 ± 258	2132 ± 380	1700 ± 398	1105 ± 203	1794 ± 195	471 ± 472
NFs (cells mL^−1^)	4805 ± 1381	3773 ± 708	2964 ± 444	2481 ± 219	2846 ± 516	1510 ± 465
*M*./PPE (%)	16.5% ± 9.2%	8.8% ± 9.2%	6.7% ± 3.9%	16.9% ± 3.5%	10.6% ± 7.8%	25.3% ± 12.8%
*M*./PNFs (%)	18.9% ± 5.9%	18.7% ± 7.2%	14.4% ± 3.7%	31.7% ± 13.1%	21.8% ± 9.3%	47.0% ± 18.9%
*M*./NFs (%)	9.9% ± 4.9%	11.0% ± 4.2%	8.1% ± 1.8%	13.8% ± 6.5%	12.2% ± 6.5%	10.6% ± 2.4%

*Note*: Refer to Figure 3 for variable abbreviations.

### Environmental and spatial factors jointly shape *Micromonas* assemblages

Our research found consistent relationships between the environmental physicochemical factors with the abundance of *Micromonas* and its related microbial communities. Specifically, the abundance of *Micromonas*, PPE, PNFs and NFs were all significantly influenced by temperature (positively), water depth (negatively) and nutrients (i.e. DIP and DIN) concentration (negatively; Figure 5; Figure S8A,B,D,E). As a mixture of the photosynthetic and phagotrophic plankton, *Micromonas* appeared to show similar responses to the environment either as autotrophs or as heterotrophs. Temperature is considered one of the most fundamental determinants of biological processes, proven to significantly explain the spatiotemporal distribution and abundance of marine organisms (Caron & Hutchins, 2012). Generally, high environmental temperatures exert a strong facilitation on the trophic activities, metabolic activities and growth rates of marine plankton (Kordas et al., 2011). The broad distribution of *Micromonas* from tropical to polar seas indicates their wide ecological adaption to temperature (Foulon et al., 2008; Simon et al., 2017; Vader et al., 2015). Previous studies have demonstrated a positive relationship between the nutrient and the abundance of phytoplankton or NFs in the upper water layer (<50 m) in summer, explained by the weak wind mixing and strong stratification in the upper water reducing nutrients' availability and resulting in low NF abundance (Huang et al., 2008; Lin et al., 2014), which seems controversy to our result. Considering the whole depth profile (from surface to deep 1000 m) of the current study, the nutrient showed a positive relationship with water depth, while the abundance of the microbial communities decreased with water depth (Figure 5; Figure S7C–K). The discrepancy was probably due to the different scale of the depth with the previous studies. Indeed, the relationships between microbial abundance and nutrients (especially DIN) in the upper layer (<100 m) were also positive in this study, although not significant (*p* > 0.05, Figure S9). This might be due to the upwelling happening in some stations of the studied area. For example, high abundance of *Micromonas* was observed in the surface and DCM layers in several nearshore stations A1, A5, B1 and B5 (Figure S4A,B), near the coast or around the Taiwan shallow bank where upwellings usually happened in summer (Hong et al., 2011), cooperated with the rich nutrient that brought by upwellings mixing with the high‐temperature upper waters that promotes plankton growth, consequently increasing abundance (Chiang et al., 2014). However, although the nutrients were more abundant in the deep layers, other factors such as lower temperature and lower irradiance may limit the growth of the microbial organisms (Figure S7A,C,D). Higher irradiance exposures in the upper mixed layer may facilitate photosynthesis as well as feeding rate (Caron & Hutchins, 2012; Mansour & Anestis, 2021). However, the peak abundance of *Micromonas*, PPE, PNFs and NFs all occurred in sub‐surface water rather than surface water (Figures 3 and 4), probably related to the strong UV radiation that the surface layer was subjected to, which restricted the growth and behaviour of certain UV sensitive organisms (Conan et al., 2008; Macaluso et al., 2009; Vaulot & Marie, 1999). A similar result was found in a pan‐Arctic strain of *Micromonas* CCMP2099 that appeared to be adapted to photosynthesize at lower light irradiances (McKie‐Krisberg & Sanders, 2014). Strong photoinhibition was provoked in *Micromonas* by abrupt exposure to high light and UV radiation, with some genes decreasing or increasing in expression, indicating a photoprotective mechanism of *Micromonas* (Cuvelier et al., 2017). This could also explain the larger vertical variation of the abundance of *Micromonas* and PPE during daytime than at night (Figure 4A,B), probably due to the convergent light intensity and convective mixing at night that weaken the vertical gradients (Vaulot & Marie, 1999). Another study found a positive effect of dust inputs nearshore that could reduce or invert the UV radiation damage on photosynthesis and the growth of picoplankton (González‐Olalla et al., 2017), which provides a possible reason for the higher abundance of microbial communities in the nearshore habitats in this study (Figure 3). In addition, the varying distribution patterns among depths or between day and night found in the current study (Figure 4) are proved to be largely influenced by the diel rhythms of cell division and feeding activity of marine microbial plankton which are mediated by the diel variations in oceanic optical properties (Arias et al., 2020; Jacquet et al., 2001). For example, a study experimentally tested that the cell division of different marine protists preferred to occur at different times (Arias et al., 2020). An in situ feeding experiment along the diel cycle found active bacterivory of pico‐phytoflagellates in daylight hours but completely ceased to feed at night (Anderson et al., 2017). The cell size and carbon content of *Micromonas* showed variations during daytime photosynthesis and nighttime division (Cuvelier et al., 2017; DuRand et al., 2002), which could affect its diel and vertical distributions. However, there is a lack of research on the growth and cellular characteristics that reflect the physiological and ecological adaption of *Micromonas* and other microbial organisms in different water depths, which needs to be further explored.

Except for the abiotic factors that support the fundamental metabolic and growth activities of *Micromonas* and other microbial communities, the more important explanation for the higher abundance of microbial communities in the epipelagic zone was based on the coordination between phytoplankton and bacteria, as well as the predation–prey relationship between picoplankton and NFs (Chiang et al., 2014). First, the abundance of bacteria was found to be positively related to the abundance of primary producers (i.e. *Synechococcus* and PPEs), thus with Chl *a* concentration (Figure 5). Photosynthetic picoplankton was the most important contributor to biomass and production of the phytoplankton communities in summer in the subtropical Pacific Ocean and usually resulted in the maximum Chl *a* in subsurface (Ning et al., 2004). High primary productivity brings abundant dissolved and particulate organic matter, which serves as a major nutrition resource for heterotrophic bacterioplankton and thus enhances secondary production (Sherr & Sherr, 2009). Meanwhile, the significantly positive relationships between the abundance of different picoplankton (i.e. bacteria, *Synechococcus* and PPEs) and NFs (including *Micromonas* and other PNFs) may indicate a predator–prey relationship between them (Figure 5; Figure S8G,J). In addition, the strongly positive relationship between the abundance of *Micromonas* and PNFs (Figure 5; Figure S8L) may also indicate the potential predation on *Micromonas* by protists grazers, for example, mixotrophic Dinoflagellata (Orsi et al., 2018). Results of VPA further revealed the important role of the biotic interactions on the distribution of *Micromonas*, because the significant explaining environmental variables displayed to be bacteria and PNF, which tend to be the prey and potential predator of *Micromonas*, respectively (Figure 6). This was consistent with a study in a coastal current in subtropical North Pacific Ocean in summer that observed significant positive correlations among *Micromonas*, PNFs and bacterioplankton (Guo et al., 2023).

As the smallest pigmented flagellate (Sherr & Sherr, 2009), *Micromona* is well known as an important group of mixotrophic phytoflagellates that have well‐integrated chloroplasts (evolutionarily and physiologically) with consumption of particulate food (McKie‐Krisberg & Sanders, 2014; Stoecker et al., 2017). It has been suggested that the small mixotrophic phytoplankton might have less dependence on inorganic nutrients due to its capability of bacterivory, as well as its larger specific surface area that requires fewer resources from the environment to grow and reproduce (Zubkov & Tarran, 2008). Studies have identified that compared with large‐size MNFs, the smaller mixotrophic flagellates (<5 μm) account for around 80% of total flagellate bacterivory in the surface layer of oligotrophic coastal water in the northwest Mediterranean Sea (Unrein et al., 2007). They were also estimated to carry out 40%–95% of bacterivory in the epipelagic zone in the North Atlantic Ocean in summer (Zubkov & Tarran, 2008). However, another study found that the smallest PNF (2–3 μm) consumed only about 2% of the total consumption of bacteria and was considered to be primarily autotrophic in surface water in a subtropical western Pacific coastal ecosystem, although the proportion of the smallest PNF (2–3 μm) accounted for 31%–71% (average 46%) of total PNF (Tsai et al., 2011). A study in oligotrophic surface waters of the Sargasso Sea claimed that 50% of the pigmented nanoplankton were observed grazing, while the contribution of mixotrophic algae to the total PNF assemblage was less than 0.5% in the DCM layer, illustrating that PNF were more abundant in the DCM, but may be not phagotrophically active (Arenovski et al., 1995). These suggested that mixotrophy could be fairly common but also dynamics in different oceanic ecosystems (Mansour & Anestis, 2021). The potential capability of pigmented plankton to shift lifestyles into mixotrophy under certain conditions complies with the ecological and evolutionary mechanisms and processes, which makes them more competitive than pure autotrophs or heterotrophs, thus explaining why they prevail worldwide ocean and perform a considerable fraction in the entire marine plankton's community (Mansour & Anestis, 2021; Selosse et al., 2017).

Recently, more and more studies focused on the evidence of phago‐mixotrophic activity in small (≤3 μm) green algal lineages under certain conditions both in laboratory and field experiments (McKie‐Krisberg & Sanders, 2014), contributed to our understanding that mixotrophic lifestyle is often flexible to vary relying on the biotic and abiotic environmental conditions like light irradiance, temperature, nutrient and prey availability (Mansour & Anestis, 2021). For example, a general trend of an increased phagotrophic strategy with increased temperature was commonly found in PNFs (Mansour & Anestis, 2021). Moreover, the condition of high irradiance combined with inorganic nutrient limitation was observed to stimulate phagotrophy in many pigmented flagellates (Mansour & Anestis, 2021; McKie‐Krisberg & Sanders, 2014; Stoecker et al., 2017). Some Chrysophyte species were found to grow in darkness if bacteria presented as prey, while their phototrophic modes triggered as a survival strategy in the absence of abundant prey (Stoecker et al., 2017). As for *Micromonas*, a study first reported the capability of bacterivory for a *Micromonas*‐like alga from field experiments in Arctic waters and demonstrated the important contribution of phagotrophy for the organisms to survival through winter darkness and obtaining a relatively large seed population during the spring growth period (Sanders & Gast, 2012). Another field study in the Arctic found the occurrence of viable cells of *Micromonas* in the mesopelagic zone down to 1000 m depth during the polar night period, which strongly suggested the shade‐adapted property and alternative trophic modes such as phagotrophy of this photosynthetic organism survive in this depth, where the irradiance is too low for photosynthesis to cover basic cellular metabolic requirements (Vader et al., 2015). This was consistent with our result that an average of 123.56 cells mL^−1^ of *Micromonas* and 48.04% proportion of *Micromonas* in the PNF sub‐community were found in the mesopelagic zone (>200 m), although currently, we are not sure whether the presence of *Micromonas* in the deep represents a dead form or an alternative ecological niche. Another cultural experiment with the pan‐Arctic strain of *Micromonas* CCMP2099 confirmed mixotrophy and firstly examined species‐specific factors that affect feeding by this phototrophic picoeukaryote, showing that carbon fixation by photosynthesis reduced and grazing activity increased under low‐nutrient conditions in the light (Gast et al., 2014). However, in experiments in the Arctic Ocean where *Micromonas polaris* dominated the PPE, no evidence of phago‐mixotrophy was observed in any strains of this species isolated from the Arctic Ocean (Jimenez et al., 2021). These contrasting results could be due to species‐specific or environmentally dependent, which highlight the variable mixotrophic strategies of different species or clades even within the same genus, influenced to some extent by environmental factors.

Overall, in this study, the wide distribution and large proportion of *Micromonas* in the microbial community in the large scale of environmental conditions from surface to the bottom layer among the nearshore and offshore habitats suggested a broad environmental adapted spectrum of this genus. Based on the qualitative analysis in this study, we inferred that the ability to graze on bacterioplankton may have great effects on *Micromonas* assemblages, although the conditions and mechanisms that trigger the phagotrophy are still not clear. Thus, further studies need to be combined with the in situ feeding experiment and advanced genomics techniques (e.g. transcriptomics, genomics and subsequent gene‐based predictive models) to validate the phagocytotic capacity and activities of such mixotrophic taxa in complex environmental conditions.

In addition, the significant contribution of the spatial factors to *Micromonas* assemblage was also found (Figure 6; Table 1; Table S5), indicating the potential impact of dispersal limitation on *Micromonas* at the large spatial scale in this study. Reports have indicated that microorganisms were not actively and freely dispersed (Hanson et al., 2012) and their movements were promoted or restricted by complex water currents depending on their direction, speed, interconnection with other water masses or other hydrological properties (Guo et al., 2023), thus the spatial factors also exhibited an important role in shaping the spatial distribution pattern of the microbial community (Hanson et al., 2012; Martiny et al., 2006). In this study, the vertical distribution pattern is the main characteristic probably due to the water stratification and complex water currents interconnection in summer in the studied area (Hong et al., 2011; Jan et al., 2010; Li et al., 2014). Previous studies found that the surface and subsurface water masses in the SCS imposed dispersal limitations that affected the vertical distribution of microbial communities (Guo et al., 2023). Therefore, it might be not easy for *Micromonas* or other microbial organisms to achieve vertical transportation from surface water down to the mesopelagic zone and vice versa.

## AUTHOR CONTRIBUTIONS

**Xin Guo:** Conceptualization (equal); data curation (equal); formal analysis (equal); investigation (equal); visualization (equal); writing – original draft (equal); writing – review and editing (lead). **Mengwen Pang:** Conceptualization (equal); data curation (equal); formal analysis (equal); investigation (equal); methodology (lead); resources (lead); visualization (equal); writing – original draft (equal); writing – review and editing (equal). **Xinyi Zheng:** Data curation (supporting); visualization (supporting); writing – review and editing (supporting). **Lingfeng Huang:** Conceptualization (equal); funding acquisition (lead); investigation (supporting); project administration (lead); resources (supporting); supervision (lead); writing – review and editing (equal).

## CONFLICT OF INTEREST STATEMENT

None declared.

## Supporting information


**Data S1:** Supporting Information.

## Data Availability

The data that support the findings of this study are available from the corresponding author upon reasonable request.
