# Supplementary material for: Micromonas , a small pigmented flagellate, predominates the nanoflagellate and photosynthetic picoeukaryote communities in the northern South China Sea
Source: Environ Microbiol Rep. 2024 Mar 27;16(2):e13244. doi: 10.1111/1758-2229.13244 (PMC10973551; doi:10.1111/1758-2229.13244)
Supplement: Supplementary file 1 — Data S1: Supporting Information. [file EMI4-16-e13244-s001.docx]

APPENDIX

Supplementary Text S1 | The technical details of sampling and processing the environmental parameters

Water temperature, salinity, and depth profiles were recorded *in situ* from SBE-CTD. Water samples (100 mL) used to determine the concentration of DIN and DIP were first filtered through 0.45 μm filter membranes, then placed into polyethylene bottles and stored at -20°C. After taking back to the laboratory, the water was measured by segmented flow automated colorimetry using the manufacturer’s standard procedures (San + + Automated Wet Chemistry Analyzer, The Netherlands).

Samples (500 mL) for Chl *a* were pre-filtered through a 200-μm-mesh sieve to remove large zooplankton and debris and then passed through 0.7-μm GF/F filters (Whatman, England). Chl *a* was then extracted by 10 mL of 90% acetone at 0°C for 20 h in the dark and measured using a Trilogy Fluorometer (Turner Designs, Trilogy Module: CHL-A NA).

Flow cytometry (FACSAria flow cytometer, Becton Dickinson) was applied to determine the abundance of bacteria, *Synechococcus*, and photosynthetic picoeukaryotes (PPE) (Chiang et al 2014, Zhao et al 2013). In this part, a water sample was pre-filtered through 20 μm nylon mesh, then, about 2 mL subsample was fixed with 20 μL 50% cold glutaraldehyde (0.5% final concentration) and kept for 20 – 30 min in the dark until transferred to liquid nitrogen for quick frozen and stored in a freezer at -80℃ for later analysis. Before the test, calibration beads were added to each sample and blended as an internal reference. *Synechococcus* and PPE are distinguished by the forward scatter (FSC), side scatter (SSC), and two fluorescence (red, 695 ± 20 nm; orange, 585 ± 21 nm) signals in the flow cytometry, while bacteria are identified by adding SYBR Green I (Molecular Probes) as the nucleic acid stain and resolved based on their green (530 ± 15 nm) fluorescence signal.

The sampling of NF cells followed the protocols with the steps of fixing, filtering, staining, and mounting (Granda and Anadon Alvarez 2008, Huang et al 2008, Lin et al 2013). Specifically, 50 mL pre-filtered water samples through 20-μm nylon mesh were fixed with cold glutaraldehyde [final concentration 0.1% (v/v)] and then filtered onto a 0.8-μm-pore-size black polycarbonate membrane filter (25 mm in diameter; Millipore, MA, United States) under low vacuum pressure (< 20 kpa). A 2-μm-pore-size membrane was put under the black membrane to confirm filtration uniformity. The cells on the filter were then stained with 4’6-diamidino-2-phenylindole (DAPI) at a final concentration of 10 µg mL^-1^ for 7 – 8 min when only 1 mL was left in the funnel. After completely filtered, the membrane was mounted onto a microscope slide and fixed with a cover slip using immersion oil (Sigma-Aldrich, MO, United States). NF were then grouped according to their trophic status and identified according to their fluorescence under epifluorescence microscopy (Leica DM 4500B) at 1,000× magnification: non-pigmented heterotrophic nano flagellates (HNF) and pigmented nanoflagellates (PNF). In short, cells that appeared blue under UV light were the biological cells, while PNFs were distinguished from HNFs by the presence of red or orange autofluorescence under a blue excitation light. At least 40 fields of view with more than 100 cells (PNF plus HNF) were examined for each filter to obtain reliable estimates of abundance.

Samples (500 mL) for microzooplankton (mainly ciliates) were filtered through 100 μm nylon mesh and then fixed with Lugol’s solution to a final concentration of 1.5%. Samples were mixed thoroughly and stood still for 48 h before siphoning the supernatant to concentrate to a final volume of 50 mL (Utermöhl 1958). The microorganisms were captured by FlowCAM with a 10 × magnification microscope objective lens and FC100 flow cell and the ciliates were selected artificially and counted through VisualSpreadsheet software (Poulton 2016).

Supplementary Text S2 | The detailed procedure of Fluorescent i*n situ* hybridization associated with tyramide signal application (TSA-FISH)

TSA-FISH was conducted with the steps of filtration, embedding, permeabilization, inactivation of endogenous peroxidases, hybridization, washing and equilibration, tyramide signal application, DAPI counterstaining, and mounting.

***Sample preparation***

Specifically, freshly fixed cells or frozen aliquots (limited to one freeze-thaw cycle) were collected through filtration onto 0.8 µm-pores 47 mm Polycarbonate filters (Millipore, Billerica, MA, United States), followed by embedding in 0.1% agarose for 5 minutes to minimize the cell loss and dehydrated in 80 – 96% ethanol for 10 – 20 s. Considering that *Micromonas* has no cell wall, there was no need to conduct an enzymatic permeabilization step before hybridization.

***Cell hybridization by TSA-FISH***

Samples were then incubated in 0.01 M HCl for 20 min to inactivate endogenous peroxidase and briefly rinsed in 1× PBS and Milli-Q water in sequence. Filter pieces were hybridized in the dark at 35℃ for 3 h in a mixture of 2 μL HPR-labeled probe (50 ng μL^−1^, Biomers.net GmbH, Germany) and 18 μL hybridization buffer, which consisted of X% deionized formamide (Sangon Biotech Inc., China), 0.01% (w/v) sodium dodecyl sulfate (SDS, Sangon Biotech Inc., China), 0.9 M NaCl, 20 mM Tris-HCl pH 7.4 (Sinopharm Chemical Reagent Co., Ltd, China) and 2% blocking reagent (Sangon Biotech Inc., China). Samples were then washed twice in preheated washing buffer (Y mM NaCl, 5 mM EDTA, 0.01% SDS, and 20 mM Tris-HCl pH 7.4) at 37℃ for a total of 30 min. The stringency of the hybridization conditions was optimized by adjusting the concentrations of formamide (30%, 35%, 40%, 45%, and 50%) in the hybridization buffer and NaCl in the washing buffer (Supplementary Table S2). Equilibration in TNT buffer (100 mM Tris-HCl pH 7.4, 150 mM NaCl, 0.07% (v/v) Tween 20) was done for 10 min at room temperature.

Probe hybridization was then revealed by a TSA reaction using 1× Alexa Fluor^TM^ 488 (green fluorescence) labeled Tymamide Reagent (Thermo Fisher Scientific Inc., Germany) in the dark at 37℃ for 30 min. The fluorophore-labeled tyramide was pre-mixed into the solution of 1 mL amplification buffer (1.6 M NaCl, 0.08% blocking reagent, 0.8× PBS pH 7.3 (Sinopharm Chemical Reagent Co., Ltd, China), 0.16 mM dextran sulfate (Sigma-Aldrich, USA)) and 10 μL fresh H_2_O_2_ stock solution (0.15% H_2_O_2_, 1× PBS pH 7.3). The filters were then washed twice in TNT buffer at 55°C for 20 min each, briefly rinsed in Milli-Q water, and then air dried on a clean slide.

The cellular DNA was then counterstained with DAPI (blue fluorescence) at a final concentration of 1 µg mL^-1^ for 3 – 5 min. The filters were finally mounted in glycerol medium with the volume ratio of Citifluor^TM^ AF1 (Electron Microscopy Sciences) and Vectashield (Vector Laboratories, Burlingame) at 5:1 and stored at -20°C until further microscopic analysis.

***Microscopy***

Images were acquired with epifluorescence microscopy (Leica DM 4500B) at 1,000× magnification, and the target organisms were identified with a bright green color under the blue excitation light and appeared blue when checked under UV light (Fig. 2). The exposure time and gain intensity were kept the same for all the pictures, with 200 ms and 1.5 gain for UV light and 1,000 ms and 1.5 gain for blue excitation light. At least 200 target cells or 50 microscopic fields of each sample were observed. Finally, the pictures of the positive control and false-positive control were compared to determine the optimal formamide concentration of the hybridization conditions.

Supplementary Table S1 | Detailed description of sampling information.

| **Sample ID** | **Layer**  **（Sampling depth）** | **Longitude** | **Latitude** | **Sampling Date** | **Bottom_**  **Depth (m)** | **Habitat** |
| --- | --- | --- | --- | --- | --- | --- |
| X41-S | Surface (3 m) | 118.344°E | 24.267°N | 2018/7/25 | 25 | nearshore |
| X41-DCM | DCM (10 m) | 118.344°E | 24.267°N | 2018/7/25 | 25 | nearshore |
| X41-B | Bottom (20 m) | 118.344°E | 24.267°N | 2018/7/25 | 25 | nearshore |
| X15-S | Surface (3 m) | 118.963°E | 24.159°N | 2018/7/31 | 64 | nearshore |
| X15-DCM | DCM (30 m) | 118.963°E | 24.159°N | 2018/7/31 | 64 | nearshore |
| X15-B | Bottom (59 m) | 118.963°E | 24.159°N | 2018/7/31 | 64 | nearshore |
| A1-S | Surface (3 m) | 117.842°E | 23.631°N | 2018/7/26 | 25 | nearshore |
| A1-DCM | DCM (5 m) | 117.842°E | 23.631°N | 2018/7/26 | 25 | nearshore |
| A1-B | Bottom (17 m) | 117.842°E | 23.631°N | 2018/7/26 | 25 | nearshore |
| A5-S | Surface (3 m) | 118.415°E | 23.070°N | 2018/7/26 | 37 | nearshore |
| A5-DCM | DCM (20 m) | 118.415°E | 23.070°N | 2018/7/26 | 37 | nearshore |
| A5-B | Bottom (33 m) | 118.415°E | 23.070°N | 2018/7/26 | 37 | nearshore |
| A9-S | Surface (3 m) | 118.940°E | 22.539°N | 2018/7/26 | 75 | nearshore |
| A9-DCM | DCM (42 m) | 118.940°E | 22.539°N | 2018/7/26 | 75 | nearshore |
| A9-B | Bottom (70 m) | 118.940°E | 22.539°N | 2018/7/26 | 75 | nearshore |
| B1-S | Surface (3 m) | 117.297°E | 23.201°N | 2018/7/28 | 37 | nearshore |
| B1-DCM | DCM (15 m) | 117.297°E | 23.201°N | 2018/7/28 | 37 | nearshore |
| B1-B | Bottom (32 m) | 117.297°E | 23.201°N | 2018/7/28 | 37 | nearshore |
| B5-S | Surface (3 m) | 117.812°E | 22.720°N | 2018/7/28 | 33 | nearshore |
| B5-DCM | DCM (10 m) | 117.812°E | 22.720°N | 2018/7/28 | 33 | nearshore |
| B5-B | Bottom (28 m) | 117.812°E | 22.720°N | 2018/7/28 | 33 | nearshore |
| B9-S | Surface (3 m) | 118.420°E | 22.116°N | 2018/7/28 | 1210 | offshore |
| B9-DCM | DCM (70 m) | 118.420°E | 22.116°N | 2018/7/28 | 1210 | offshore |
| B9-B | Bottom (1000 m) | 118.420°E | 22.116°N | 2018/7/28 | 1210 | offshore |
| C1-S | Surface (3 m) | 117.297°E | 23.201°N | 2018/7/30 | 36 | nearshore |
| C1-DCM/B | DCM/Bottom (31 m) | 117.297°E | 23.201°N | 2018/7/30 | 36 | nearshore |
| C8-S | Surface (3 m) | 117.782°E | 21.819°N | 2018/7/29 | 446 | offshore |
| C8-DCM | DCM (75 m) | 117.782°E | 21.819°N | 2018/7/29 | 446 | offshore |
| C8-B | Bottom (200 m) | 117.782°E | 21.819°N | 2018/7/29 | 446 | offshore |
| C11-S | Surface (3 m) | 117.782°E | 21.819°N | 2018/7/29 | 2321 | offshore |
| C11-DCM | DCM (80 m) | 117.782°E | 21.819°N | 2018/7/29 | 2321 | offshore |
| C11-B | Bottom (1000 m) | 117.782°E | 21.819°N | 2018/7/29 | 2321 | offshore |
| M3-Daytime-1 | 2 m | 119.083°E | 21.833°N | 2018/8/20 | 1991 | offshore |
| M3-Daytime-2 | 25 m | 119.083°E | 21.833°N | 2018/8/20 | 1991 | offshore |
| M3-Daytime-3 | 50 m | 119.083°E | 21.833°N | 2018/8/20 | 1991 | offshore |
| M3-Daytime-4 | 75 m (DCM) | 119.083°E | 21.833°N | 2018/8/20 | 1991 | offshore |
| M3-Daytime-5 | 100 m | 119.083°E | 21.833°N | 2018/8/20 | 1991 | offshore |
| M3-Daytime-6 | 200 m | 119.083°E | 21.833°N | 2018/8/20 | 1991 | offshore |
| M3-Daytime-7 | 500 m | 119.083°E | 21.833°N | 2018/8/20 | 1991 | offshore |
| M3-Night-1 | 2 m | 119.083°E | 21.833°N | 2018/8/20 | 1991 | offshore |
| M3-Night-2 | 25 m | 119.083°E | 21.833°N | 2018/8/20 | 1991 | offshore |
| M3-Night-3 | 50 m | 119.083°E | 21.833°N | 2018/8/20 | 1991 | offshore |
| M3-Night-4 | 75 m (DCM) | 119.083°E | 21.833°N | 2018/8/20 | 1991 | offshore |
| M3-Night-5 | 100 m | 119.083°E | 21.833°N | 2018/8/20 | 1991 | offshore |
| M3-Night-6 | 200 m | 119.083°E | 21.833°N | 2018/8/20 | 1991 | offshore |
| M3-Night-7 | 500 m | 119.083°E | 21.833°N | 2018/8/20 | 1991 | offshore |

Supplementary Table S2 | List of the probe and specificity controls used in this study, with the stringency parameters for the hybridization (formamide %) and washing (NaCl concentration) steps. ^a^ CCMP: Provasoli-Guillard National Center for Culture of Marine Phytoplankton, USA; SAG: The Culture Collection of Algae at the University of Göttingen, Germany.

| Probe | Specificity Control | | | | | Formamide (%)  Tested | NaCl (M)  Tested |
| --- | --- | --- | --- | --- | --- | --- | --- |
|  | Type | Strain ^a^ | Mismatch |  | Accession |  |  |
| Micro 01 | (+)pCtrl | *Micromonas pusilla*  CCMP154 | 0 |  | AY954994 | 30,35,**40**,45,50 | 1.6,1.05,**0.675,**0.4,0.225 |
|  | (–) pCtrl | *Chlorella sphaerica*  SAG11.88 | 2 |  | AJ416105 | 30,**35**,40,45,50 | 1.6,**1.05**,0.675,0.4,0.225 |

(+)pCtrl, positive probe control; (–)pCtrl, false-positive probe control. **Bold** means validated optimal formamide concentrations in the hybridization buffer and the corresponding NaCl concentrations in the washing buffer.

Supplementary Table S3 | *In-silico* specificity of probe Micro 01.

| Probe | 0 mismatch | |  | 1 mismatch | |  | 2 mismatches | |  | 3 mismatches | | | |
| --- | --- | --- | --- | --- | --- | --- | --- | --- | --- | --- | --- | --- | --- |
|  | TS | Total. S |  | TS | Total. S |  | TS | Total. S |  | TS | Total. S | Total.TS | TS/ Total.TS |
| Micro 01 | 112 | 112 |  | 350 | 750 |  | 358 | 3,795 |  | 358 | 16,667 | 368 | 97.28% |

TS, the number of matched target sequences; Total.S, the number of total matched sequences; Total.TS, the number of total target sequences (including 10 unmatched target sequences).

Supplementary Table S4 | Ten unmatched target organisms with 3 central mismatches.

| **Probe** | **Organism** | **Accession** |
| --- | --- | --- |
| Micro 01 | Uncultured eukaryote | GU825479 |
|  | Uncultured marine eukaryote | EU371170 |
|  | Uncultured marine eukaryote | EU371171 |
|  | Uncultured marine eukaryote | EU371172 |
|  | Uncultured marine eukaryote | EU371180 |
|  | Uncultured marine eukaryote | EU371182 |
|  | Uncultured marine eukaryote | HM581746 |
|  | Uncultured Chlorophyta | JX840897 |
|  | Uncultured Chlorophyta | JX840899 |
|  | Uncultured Chlorophyta | JX840928 |

Supplementary Table S5 | The backward selection establishes the best linear model of the relationship between *Micromonas* abundance and variable factors, with *Micromonas* abundance being the dependent variable. The *P*-values and Akaike Information Criterion (AIC) for each variable in all steps were presented. The variable (marked by *) with the highest *P*-value and lowest AIC was removed in every step. Note that the fourth eigenvector (PCNM4) was used to represent the spatial variable that accounts for the spatial autocorrelations and dispersal effects because it was tested to be the only spatial factor that was always significant in each step of backward selection. Significant codes: ***, *P* < 0.001; **, *P* < 0.01; *, *P* < 0.05; ^+^, *P* < 0.1. Refer to Fig. 3 for variable abbreviations.

| **Model selection** | **Independent variable** | ***P***-value | **AIC** |
| --- | --- | --- | --- |
| Selection step 1 | Depth* | 0.894 | -70.343 |
|  | Salinity | 0.772 | -70.241 |
|  | DIN | 0.668 | -70.088 |
|  | PPE | 0.354 | -69.057 |
|  | *Synechococcus* | 0.247 | -68.321 |
|  | Ciliates | 0.134 | -66.928 |
|  | Chl *a* | 0.104 | -66.321 |
|  | Bacteria | 0.044* | -64.178 |
|  | HNF | 0.028* | -62.933 |
|  | PCNM4 | 0.004** | -57.986 |
|  | PNF | < 0.0001*** | -42.291 |
| Selection step 2 | Salinity* | 0.643 | -72.028 |
|  | DIN | 0.579 | -71.891 |
|  | PPE | 0.349 | -71.057 |
|  | *Synechococcus* | 0.210 | -70.038 |
|  | Ciliates | 0.121 | -68.806 |
|  | Chl *a* | 0.097_+_ | -68.304 |
|  | Bacteria | 0.026* | -65.042 |
|  | HNF | 0.025* | -64.931 |
|  | PCNM4 | 0.003** | -59.065 |
|  | PNF | < 0.0001*** | -43.845 |
| Selection step 3 | DIN* | 0.561 | -73.551 |
|  | PPE | 0.395 | -73.008 |
|  | *Synechococcus* | 0.133 | -70.839 |
|  | Ciliates | 0.130 | -70.798 |
|  | Chl *a* | 0.093_+_ | -70.043 |
|  | Bacteria | 0.026* | -67.042 |
|  | HNF | 0.011* | -64.789 |
|  | PCNM4 | 0.002** | -60.720 |
|  | PNF | < 0.0001*** | -44.615 |
| Selection step 4 | PPE* | 0.384 | -74.522 |
|  | Ciliates | 0.137 | -72.550 |
|  | *Synechococcus* | 0.124 | -72.341 |
|  | Chl *a* | 0.100 | -71.873 |
|  | Bacteria | 0.026* | -68.800 |
|  | HNF | 0.011* | -66.751 |
|  | PCNM4 | 0.002** | -62.719 |
|  | PNF | < 0.0001*** | -44.261 |
| Selection step 5 | Ciliates | 0.194 | -74.312 |
|  | *Synechococcus* | 0.070_+_ | -72.227 |
|  | Bacteria | 0.035* | -70.712 |
|  | HNF | 0.014* | -68.610 |
|  | PCNM4 | 0.003** | -64.716 |
|  | Chl *a* | 0.001** | -62.691 |
|  | PNF | < 0.001*** | -42.851 |


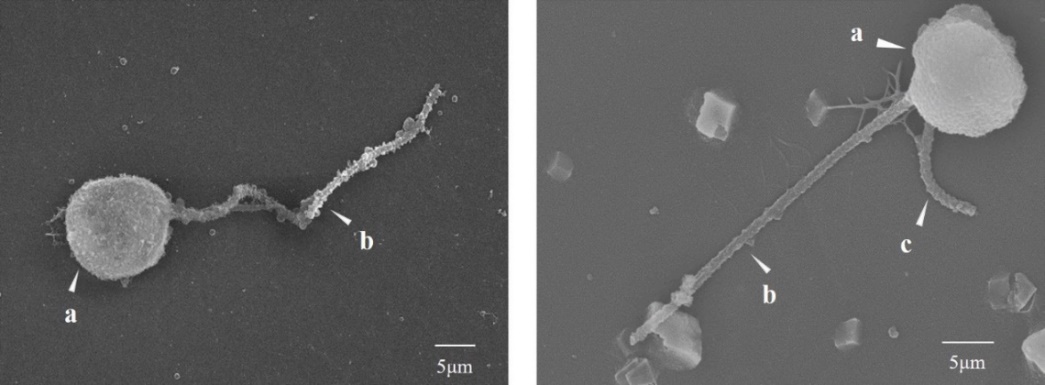


Supplementary Figure S1 | Observation of Micromonas pusilla, CCMP1545 under the scanning electron microscope (a is the head; b is the long flagella; c is the short flagella).


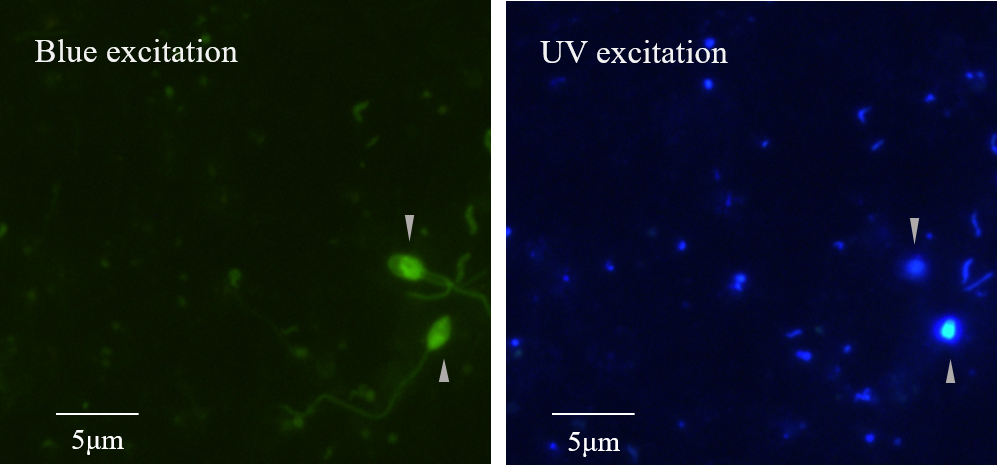


Supplementary Figure S2 | Images of *Micromonas* in environmental samples under the fluorescence microscope, which were processed by TSA-FISH with the optimal formamide concentration in hybridization buffer at 40%. Target cells dyed with Alexa Fluor 488^TM^ and DAPI in TSA-FISH, performing bright green and blue under the epifluorescence microscopy, respectively. The white arrows indicate the target organisms. Scale bars indicate 5 µm.


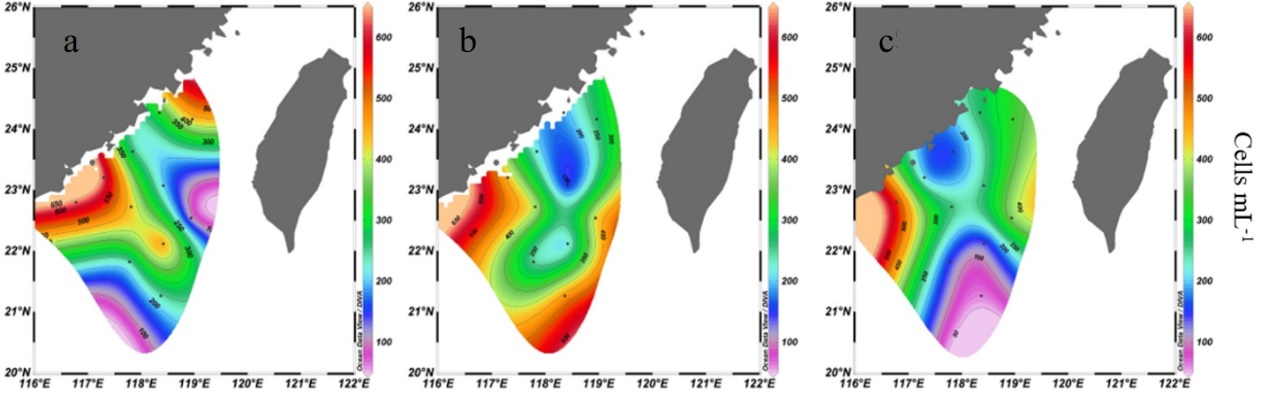


Supplementary Figure S3 | Horizontal distribution of *Micromonas* in surface (a), DCM (b), and bottom (c) layer of 11 large-scale-observation stations in the northern South China Sea.


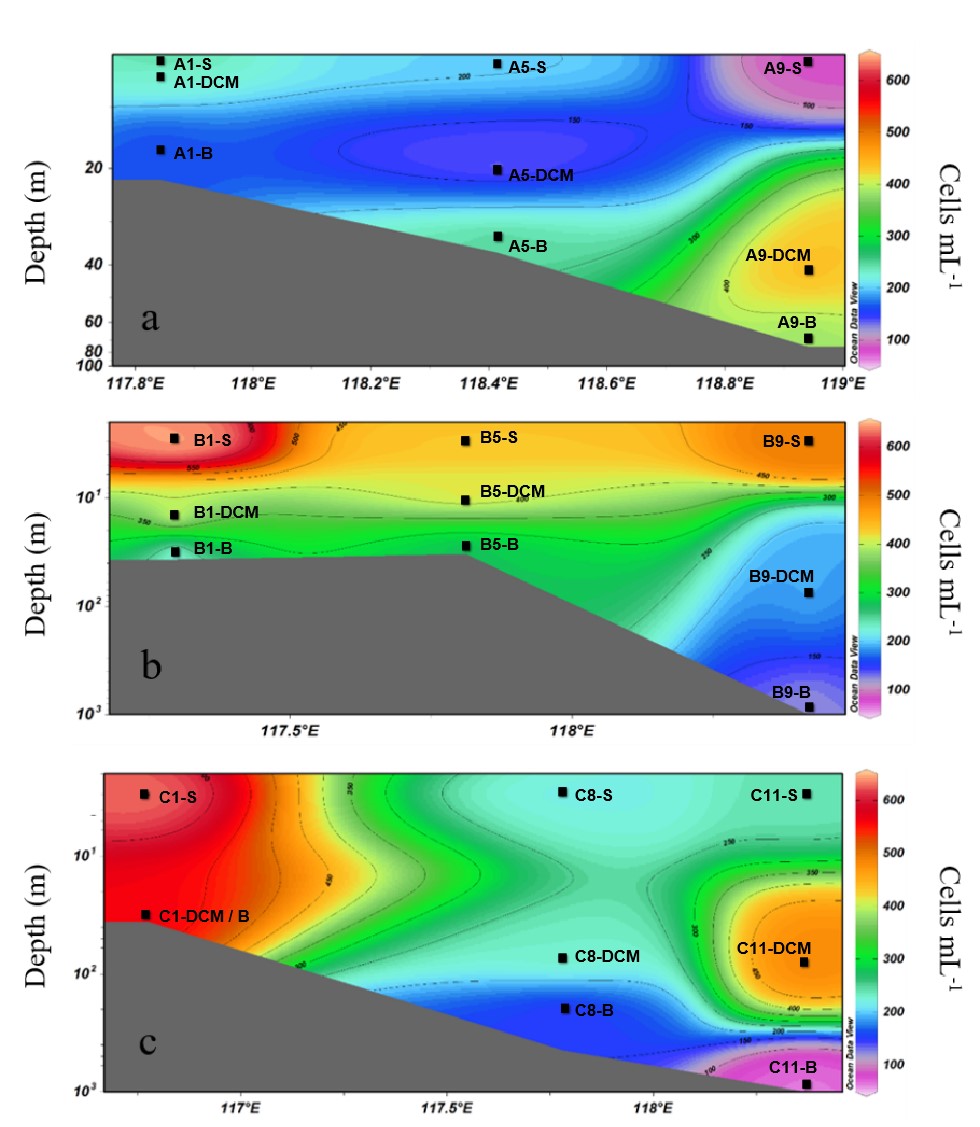


Supplementary Figure S4 | Vertical and horizontal distribution of *Micromonas* abundances (cells mL^−1^) along the transect A (a), B (b), and C (c) in the northern South China Sea. Black dots correspond to sampling points.


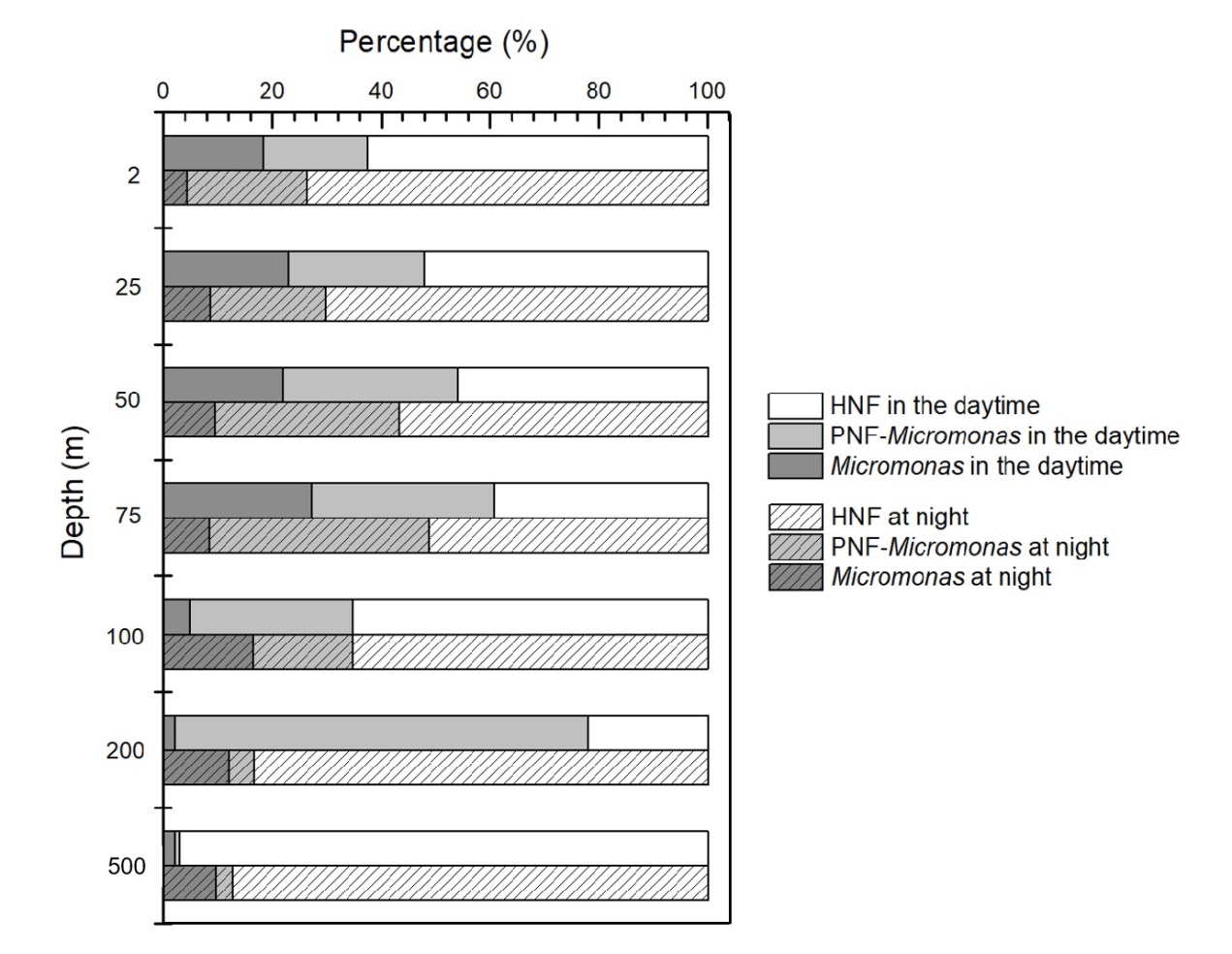


Supplementary Figure S5 | The proportion of *Micromonas* in NFs community in a different layer of M3 station in the daytime and at night. PNFs-*Micromonas* represent the non-*Micromonas* pigmented nanoflagellates.


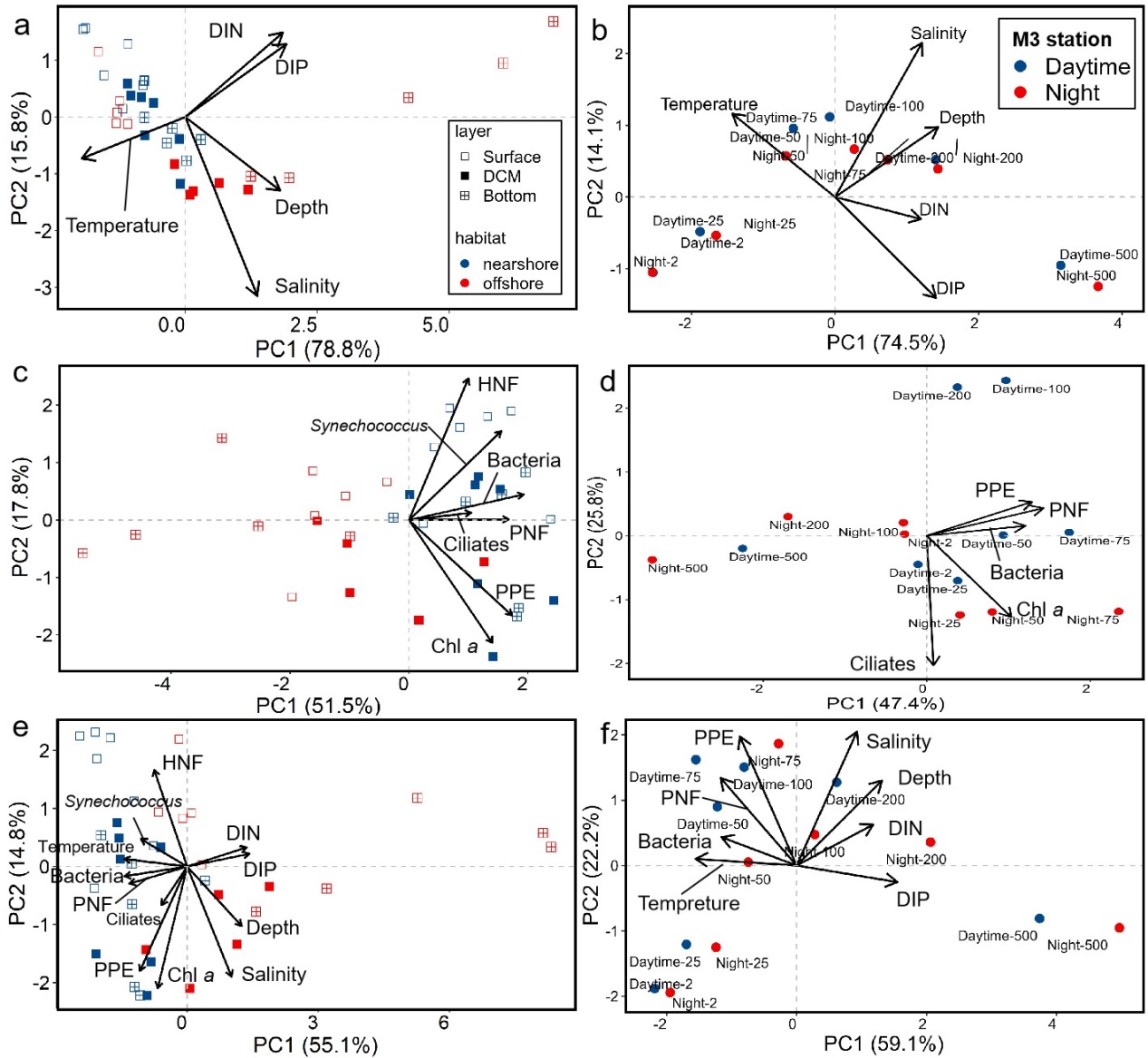


Supplementary Figure S6 | Ordination biplots of the principal component analysis (PCA) of the abiotic factors (a, b), biotic factors (c, d), and all environmental variables (e, f) for the large-scale-observation stations (left panels) and diel-continuous-observation station M3 (right panels). All environmental factors were log(x+1) transformed. The explanatory variables factors selected in the analyses are all significant (*P* < 0.05). Refer to Fig. 3 for variable abbreviations.


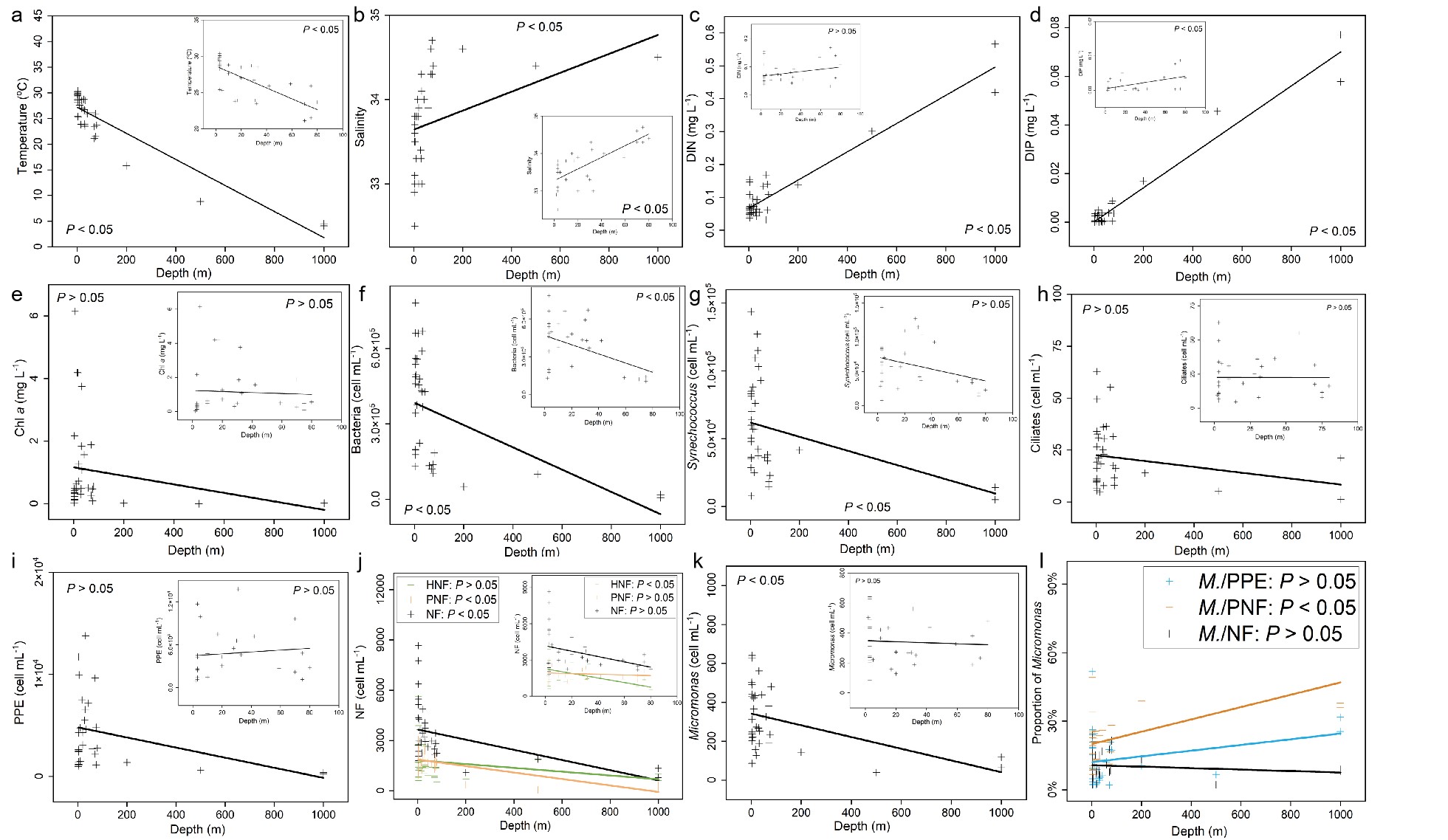


Supplementary Figure S7 | Vertical distributions of the environmental abiotic factors (a-d), biotic factors (e-j), abundance of *Micromonas* (k), and the proportions of *Micromonas* in the related groups (l) with the increase of sampling depth. The Lines represent linear fits between each variable and depth. The attached small figures in each panel display the relationships within the upper euphotic zone from the surface to 100 m depth.


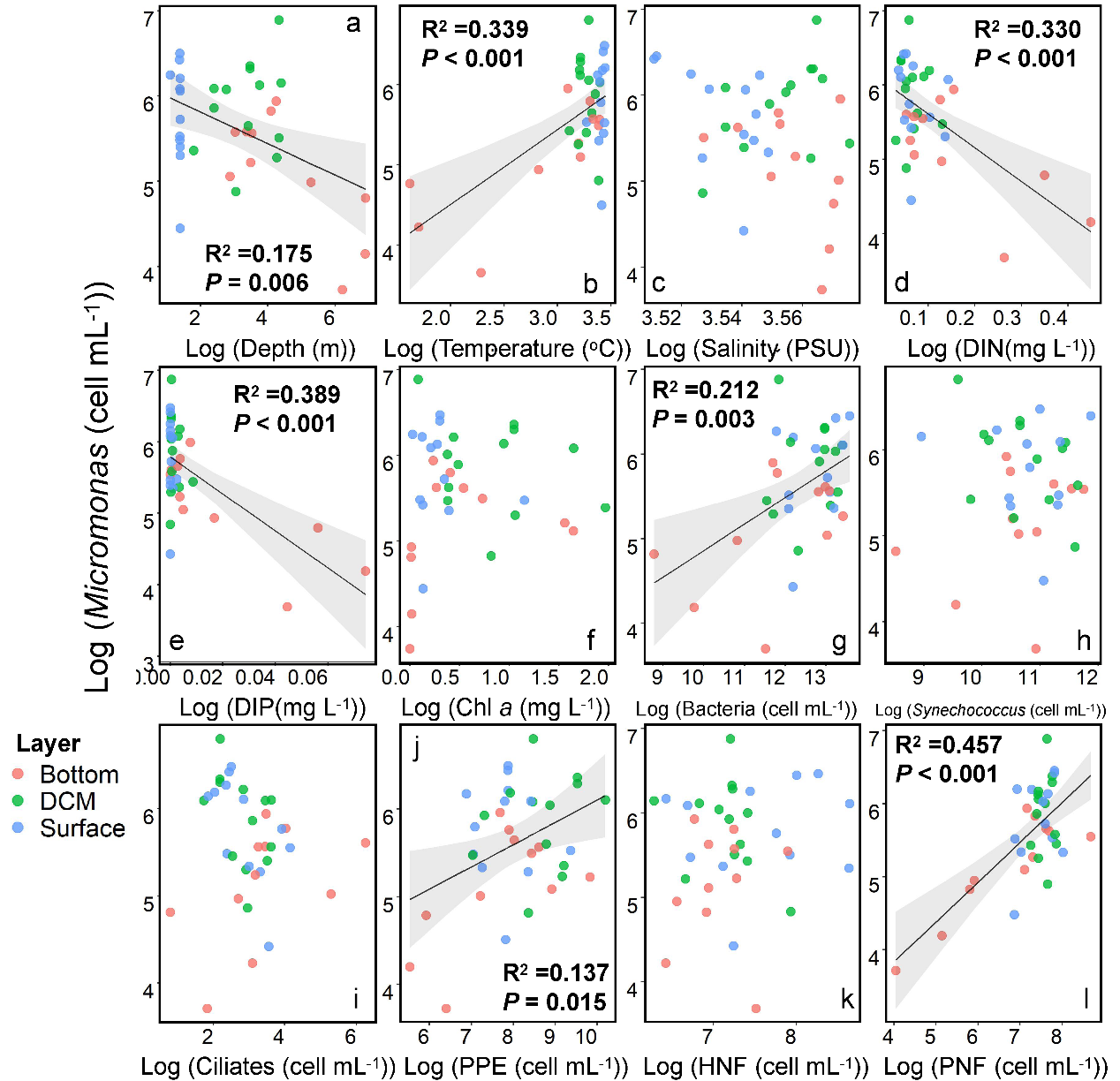


**Supplementary Figure S8 |** The linear relationships between the abundance of *Micromonas* and environmental variables, including abiotic factors (a-d) and biotic factors (e-l). All the variables are log(x+1) transformed. The solid lines represent linear fits between each pair with a *P*-value < 0.05. The grey-shaded area around the black solid line indicates the confidence interval of the regression line (Linear Model). The colors of the dots represent different layers. Refer to Fig. 3 for variable abbreviations.


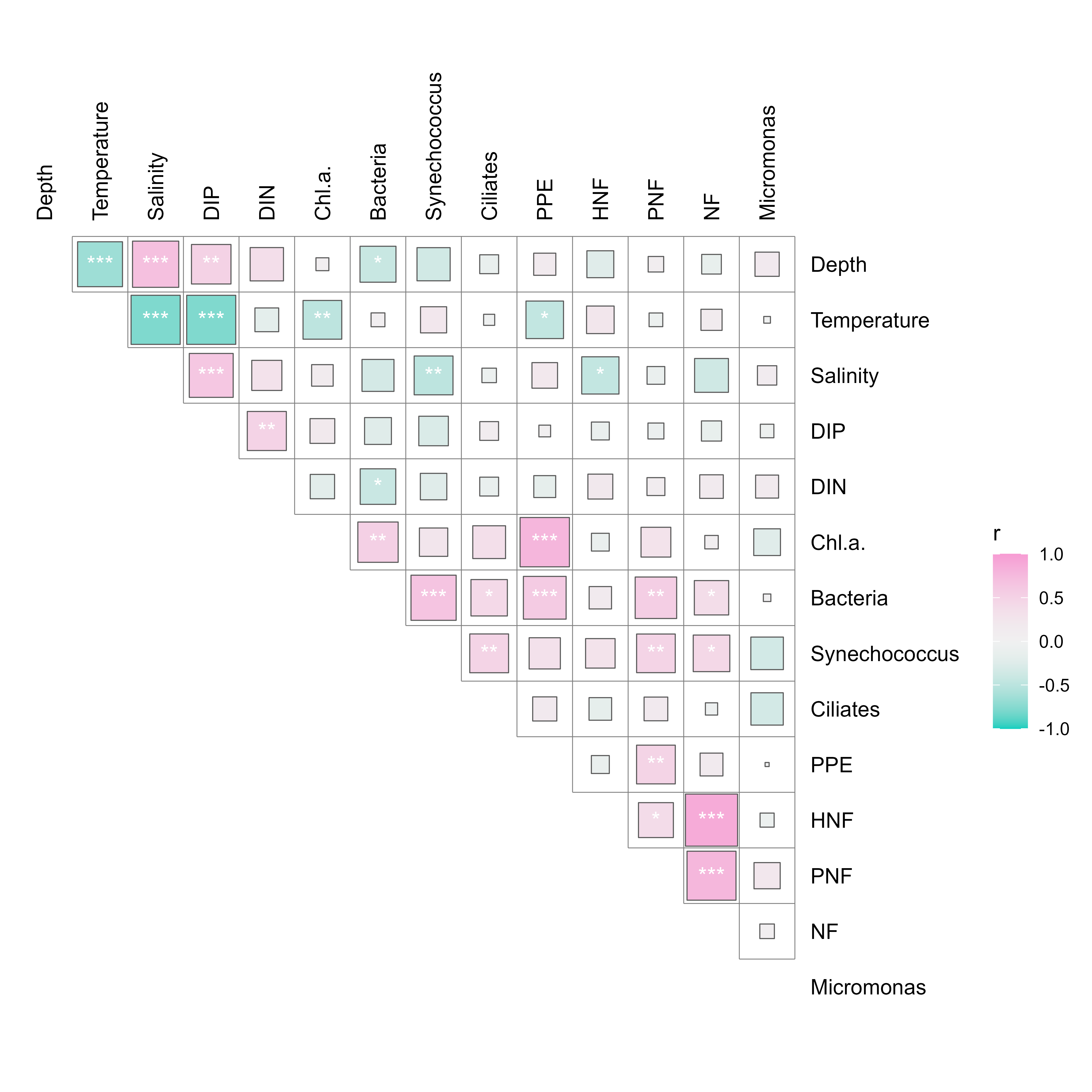


Temperature

Salinity

DIP

DIN

Chl *a*

Bacteria

*Synechococcus*

Ciliates

PPE

PNF

HNF

*Micromonas*

NF

Temperature

Salinity

Depth

DIP

DIN

Chl *a*

Bacteria

*Synechococcus*

Ciliates

PPE

PNF

HNF

NF


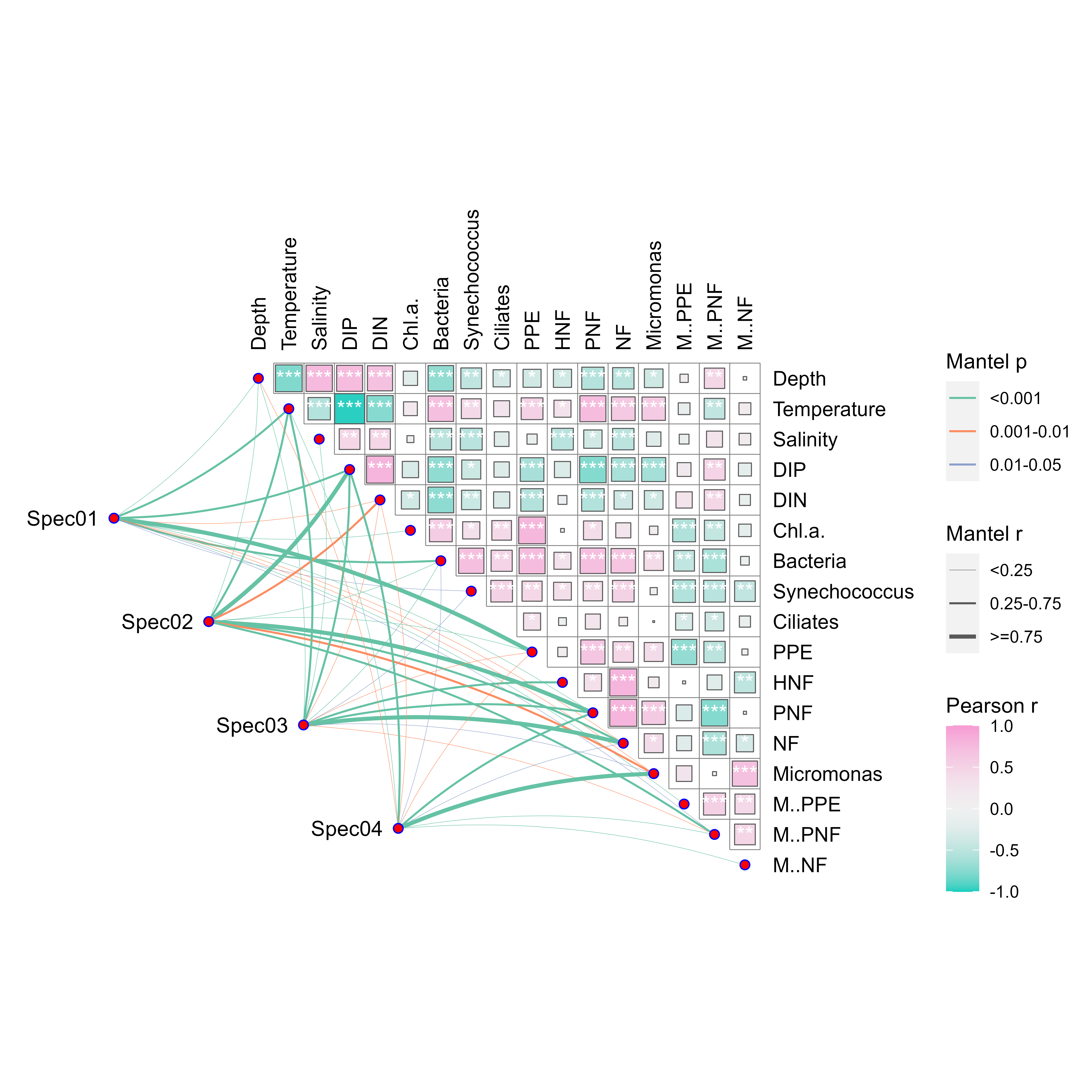


Supplementary Figure S9 | Environmental drivers (including abiotic and biotic factors) of the abundance of *Micromonas* in the upper layer (<100 m) in the northern South China Sea. Pairwise comparisons of environmental variables are shown with a color gradient denoting Pearson’s correlation coefficient. All the variables are log(x+1) transformed. Significant relationships labeled with asterisk (*, *P* < 0.05; **, *P* < 0.01; ***, *P* < 0.001) based on 9999 permutations were shown. Refer to Fig. 3 for variable abbreviations.
